# Overexpression of the *WOX* gene *STENOFOLIA* improves biomass yield and sugar release in transgenic grasses and display altered cytokinin homeostasis

**DOI:** 10.1371/journal.pgen.1006649

**Published:** 2017-03-06

**Authors:** Hui Wang, Lifang Niu, Chunxiang Fu, Yingying Meng, Dajun Sang, Pengcheng Yin, Jinxia Wu, Yuhong Tang, Tiegang Lu, Zeng-Yu Wang, Million Tadege, Hao Lin

**Affiliations:** 1 Biotechnology Research Institute, Chinese Academy of Agricultural Sciences, Beijing, China; 2 Department of Plant and Soil Sciences, Institute for Agricultural Biosciences, Oklahoma State University, 3210 Sam Noble Parkway, Ardmore, OK, United States of America; 3 Forage Improvement Division, Samuel Roberts Noble Foundation, 2510 Sam Noble Parkway, Ardmore, OK, United States of America; 4 Plant Biology Division, Samuel Roberts Noble Foundation, 2510 Sam Noble Parkway, Ardmore, OK, United States of America; University of California Berkeley, UNITED STATES

## Abstract

Lignocellulosic biomass can be a significant source of renewable clean energy with continued improvement in biomass yield and bioconversion strategies. In higher plants, the leaf blade is the central energy convertor where solar energy and CO_2_ are assimilated to make the building blocks for biomass production. Here we report that introducing the leaf blade development regulator *STENOFOLIA* (*STF*), a WOX family transcription factor, into the biofuel crop switchgrass, significantly improves both biomass yield and sugar release. We found that *STF* overexpressing switchgrass plants produced approximately 2-fold more dry biomass and release approximately 1.8-fold more solubilized sugars without pretreatment compared to controls. The biomass increase was attributed mainly to increased leaf width and stem thickness, which was also consistent in *STF* transgenic rice and *Brachypodium*, and appeared to be caused by enhanced cell proliferation. STF directly binds to multiple regions in the promoters of some cytokinin oxidase/dehydrogenase (*CKX*) genes and represses their expression in all three transgenic grasses. This repression was accompanied by a significant increase in active cytokinin content in transgenic rice leaves, suggesting that the increase in biomass productivity and sugar release could at least in part be associated with improved cytokinin levels caused by repression of cytokinin degrading enzymes. Our study provides a new tool for improving biomass feedstock yield in bioenergy crops, and uncovers a novel mechanistic insight in the function of *STF*, which may also apply to other repressive *WOX* genes that are master regulators of several key plant developmental programs.

## Author summary

The leaf blade of higher plants serves as a solar panel in which it captures solar energy and carbon dioxide to produce chemical energy in the process of photosynthesis. Thus, the ultimate source of food and feed for most heterotrophic organisms, including humans, comes from the photosynthetic activity of leaves. Plant biomass also promises to be a significant source of transportation fuels which could alleviate some of the stigma of environmental pollution, scarcity and finite resources associated with gasoline. Wider leaf blade may be expected to increase biomass yield and overall plant growth due to larger photosynthetic surface area. Here we introduced a leaf blade outgrowth regulatory factor, *STF*, from eudicot species into three grasses, switchgrass, *Brachypodium* and rice, and found that the transgenic plants formed much wider leaves compared to controls. Consequently, transgenic switchgrass plants produced approximately two-fold more total biomass and solubilized sugars without acid pretreatment, demonstrating a novel approach for improving biomass feedstock properties. We also show that the transgenic rice seedlings accumulate the phytohormone cytokinin at higher levels, uncovering a novel mechanism that links *STF* activity to cytokinin homeostasis. Our work will significantly advance understanding of the mechanistic function of *WOX* genes in plant development.

## Introduction

Plant biomass is an abundant source of renewable energy and biomaterials, and sustainable lignocellulosic fuel ethanol production from biomass feedstocks has a great potential to be exploited as an alternative energy source to meet increasing energy demands worldwide [1]. The United States, for example, has projected to meet approximately 30% of its energy demands by 2030 from such renewable sources [2]. However, apart from the logistics of biomass transportation and processing, significant challenges still persist in biomass feedstock yield and saccharification efficiency. Plant cell wall, the most abundant plant biomass, is composed of cellulose and hemicellulose matrix polysaccharides copolymerized with a phenolic polymer lignin forming a complex crosslink [3–5]. This makes the polysaccharides recalcitrant to enzymatic digestion to soluble sugars (saccharification) for microbial conversion to biofuels [6]. Current biomass conversion technologies utilize acid pretreatment at high temperatures to break apart the lignin polymer and expose the polysaccharides. Such a pretreatment, in addition to cost and environmental pollution, negatively impacts downstream microbial fermentation, reducing the market competitiveness of biofuels. Accordingly, enhancing biomass yield and saccharification efficiency has become a major research focus for the genetic improvement of bioenergy crops. Switchgrass is one of the dedicated bioenergy crops in the USA [7] and research has been intensified in the last few years to increase yield and reduce lignin content in an attempt to improve its feedstock properties [8–10].

The leaf blade is the energy powerhouse of plants where solar energy and CO_2_ are assimilated to produce the chemical energy used in food, feed and biofuels. Since the leaf blade essentially serves as a solar panel in capturing sunlight, its size and design should have a significant bearing on biomass productivity through increasing photosynthetic efficiency [11–13]. Redesigning the leaf blade is, therefore, potentially a major target for improving biomass feedstock yield. Blade outgrowth is regulated by several antagonistically acting polarity factors that are exclusively expressed either on the upper (adaxial) or lower (abaxial) side of the leaf at least in eudicots. These factors include *AS1*, *AS2*, *HD ZIP Ⅲ* genes and *tasiR-ARF*s on the adaxial side and *KAN*, *FIL*, *YAB*, *miRNA165/6* and *ARF3/4* on the abaxial side in *Arabidopsis* and are required for polarity specification and cell differentiation in their respective domains [14–18]. Extensive studies in *Arabidopsis* over the past two decades revealed that the combined action of polarity factors and multiple phytohormones is required for the establishment and growth of a determinate bilaterally symmetrical leaf blade from undifferentiated pluripotent cells of the shoot apical meristem (SAM). The leaf marginal meristem (blastozone) has long been recognized as the site of cell proliferation for lateral expansion of the leaf blade after recruitment of leaf founder cells from the SAM and establishment of the leaf primordium [19–21]. However, leaf growth in the proximal-distal (length) direction appears to be to some extent independent from growth in the medial-lateral (width) direction as demonstrated by several genetic mutants affected only in leaf width [22] including the *bladeless lam1* mutant of *Nicotiana sylvestris* and the *stenofolia* (*stf*) mutant of *Medicago truncatula*.

In monocots, *Wavy auricle in blade 1* (*Wab1*) and *Liguleless narrow-R* (*Lgn-R*) mutants in maize [23–25] and *narrow leaf 1* (*nal1*) in rice [26] display narrow leaf blades but defects in these mutants appear to include proximal-distal growth as well. On the other hand, the maize, *narrowsheath1* (*ns1*) and *narrowsheath2* (*ns2*) double mutant has a very narrow leaf blade affected in medial-lateral growth [27] without significant defect in leaf length. *ns1* and *ns2* are duplicate *WUSCHEL*-related homeobox (WOX) transcription factors homologous to *Arabidopsis WOX3/PRS* [28]. Mutations in homologous genes, *nal2* and *nal3* double, also cause narrow leaves in rice [29, 30]. The *nal2/3* double mutant displays a pleiotropic phenotype including narrow-curly leaves, more tillers, fewer lateral roots, open spikelets and narrow-thin grains [30], indicating a widespread effect on overall plant development. Auxin transport related genes are found to be altered in expression in the *nal2/3* double mutant [30], and the OsWOX3A protein, encoded by *NAL2/3*, is shown to be involved in negative feedback regulation of GA biosynthesis [31]. Transcriptome analysis in a laser dissected *ns1/2* mutant shoot apex in maize also identified changes in hormonal signaling pathways including auxin and jasmonate [32]. However, the actual molecular mechanisms for the function of these *WOX* genes in blade lateral outgrowth remains unclear.

We cloned the *stf* and *lam1* mutants previously and shown that they are caused by mutations in the same gene that encodes for a putative WUSCHEL-related homeobox (WOX) transcription factor [33] similar to petunia *MAW* and *Arabidopsis WOX1* [34]. *STF* is expressed at the adaxial-abaxial juxtaposition of the growing leaf primordium that includes the leaf margins and the middle mesophyll, and critically regulates blade outgrowth by activating cell proliferation [33], which was confirmed by *Arabidopsis WOX1* [35], suggesting a *WUS* like function in leaf margins. STF promotes cell proliferation through a transcriptional repression activity [36, 37] that involves the corepressor TOPLESS (MtTPL) [38]. Transcriptional repression activity is a requirement for STF’s blade outgrowth function, and multiple phytohormones including auxin and cytokinin have been proposed to be involved in STF function [33, 39], but the connection between transcriptional repression and activity of hormones has not been firmly established.

Here we report that ectopic expression of *STF* in three monocot species, switchgrass, *Brachypodium* and rice leads to improvement in biomass yield. We show that STF directly binds to several regions in the promoters of cytokinin oxidase/dehydrogenase genes and represses their transcription allowing accumulation of active cytokinin pools, highlighting a novel mechanism for WOX-mediated cell proliferation via transcriptional repression.

## Results

**Fig 1:**
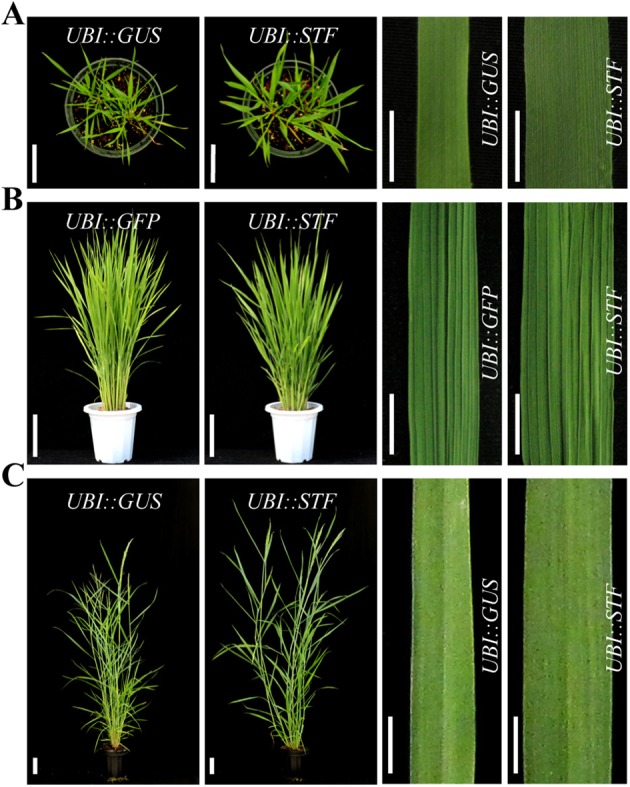
*STF* overexpression universally increases leaf blade width in grasses. (A) Phenotypes of *STF* overexpressing transgenic *Brachypodium* and *GUS* expressing control. The right panel shows a close-up of control and *STF* transgenic leaves from the same position. Bars = 5 cm for plants and 1 cm for leaves. (B) Phenotypes of *STF* overexpressing rice and *GFP* transgenic rice used as control with a close-up of equivalent leaves shown on the right. Bars = 10 cm for plants and 1 cm for leaves. (C) Phenotype of *STF* overexpressing switchgrass and *GUS* expressing control, the right panel showing close-up of leaves from the same positions. Bars = 10 cm for plants and 1 cm for leaves. All the three *STF* transgenic grasses showed significantly broader leaves than their respective controls.

The *Medicago WOX* gene *STF* is a master regulator of plant growth and development required for leaf blade outgrowth, leaf vascular patterning, stem thickness, inflorescence fusion, petal expansion, ovule development and female fertility [33]. Although *STF*-like sequencers are conserved in eudicots and the early diverging angiosperm *Amborella trichopoda* [40], obvious *STF* homologues have not been identified in monocotyledonous plants [33, 38]. We wondered whether *STF* could be used to modify leaf size and thereby increase vegetative biomass in grasses. To test the hypothesis that the *STF* gene could be used to manipulate leaf size and improve biomass yield in grasses, we introduced the full-length *STF* CDS into the bioenergy crop switchgrass (*Panicum virgatum* L.) as well as two grass models *Brachypodium* and rice under the control of the maize *UBIQUITIN* (*UBI*) promoter by using *Agrobacterium*-mediated transformation. Meanwhile, we generated *UBI*::*GUS* and *UBI*::*GFP* transformants as controls. We observed that *STF* overexpressing transgenic lines in *Brachypodium*, rice and switchgrass showed significant morphological changes in leaf blade expansion compared with control plants (Fig 1). Each of the *STF* transformants showed wider leaf blade and thicker stem than controls but also displayed plant height phenotypes depending on *STF* expression levels. In general, increasing *STF* expression levels were correlated with increasing phenotypic severity. While modest level of *STF* expression promoted leaf expansion and stem thickness in all the three species, high level of expression drastically reduced plant height, and caused leaf curling and scattered deformation on the leaf vasculature although the blade still remained wider than controls (Fig 1 and S1 and S2 Figs).

**Fig 2:**
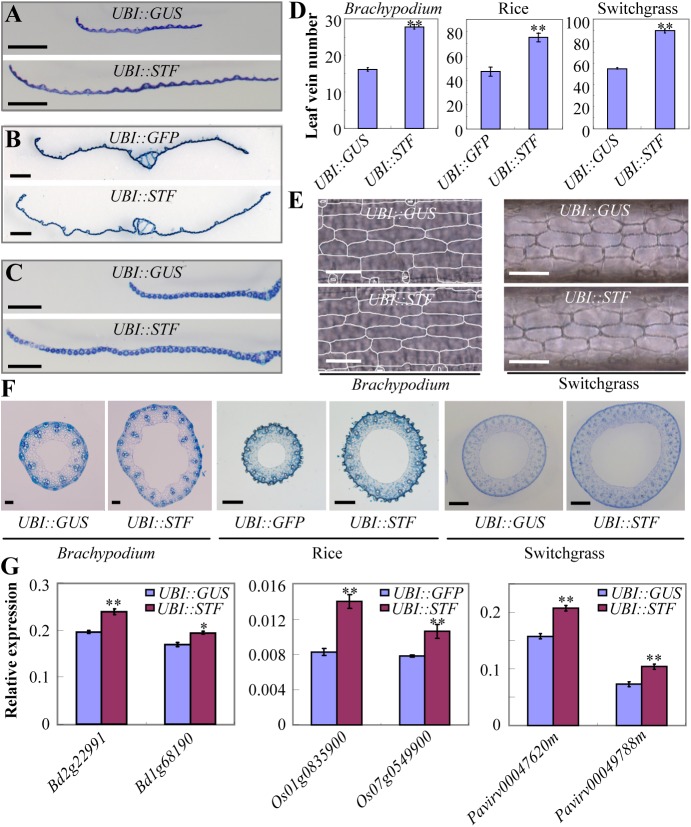
Ectopic expression of *STF* in grasses leads to significant increase in plant size primarily through promoting cell proliferation. (A-C) Cross section of flag leaves of the control and *STF* overexpressing *Brachypodium* (A), rice (B) and switchgrass (C). Bars = 1 mm. (D) Comparison of leaf vein number of the control and *STF* transgenic *Brachypodium*, rice and switchgrass. Bars represent means ± SE (n = 7 plants). (E) Micrographs of cleared leaves of *STF* transgenic *Brachypodium* and switchgrass. Bars = 100 μm. (F) Cross section of internode Ⅱ (for rice and *Brachpodium*) and internode Ⅲ (for switchgrass) of *STF* transgenic *Brachypodium*, rice and switchgrass. Bars = 100 μm in *Brachypodium*, 1mm in rice and switchgrass. (G) Transcript level of *Histone H4* in *STF* overexpressing *Brachypodium*, rice and switchgrass compared to controls. Bd, *Brachypodium distachyon*; Os, *Oryza*. *Sativa*; Pavirv, *Panicum virgatum*. Bars represent means ± SE of three technical replicates, two biological replicates. The asterisks indicate significant differences (* p<0.05, ** p < 0.01, Student t-test).

In agreement with the morphological changes observed above, histological analysis showed that the cell number as well as the number of vascular bundles were significantly increased in *STF* overexpressing leaves and culms in all the three monocot plants (Fig 2A–2D and 2F). Cross section through the leaf blade or stem indicated that the number of veins was significantly increased (p < 0.01) in the leaf (Fig 2A–2D) and stems were significantly thicker (Fig 2F) in all the transgenic lines compared with their respective controls. Control plants were transformed with *UBI*::*GUS* in *Brachypodium* and switchgrass, and with *UBI*::*GFP* in rice using the same vector pMDC32. Measurement of leaf width in transgenic switchgrass was consistent with the number of veins in quantifying blade lateral expansion (S1 Table). However, examination of leaf epidermal cells in *UBI*::*STF* transformants in *Brachypodium* and switchgrass showed that the cell size was not obviously changed (Fig 2E), suggesting that the wider leaf blade and thicker stem phenotypes were mainly caused by enhanced cell proliferation. Consistent with this, quantitative real time PCR (qRT-PCR) analysis showed that the transcript level of the cell division marker *Histone H4* was significantly increased in *STF* overexpression lines in all the three species (Fig 2G). These results together indicate that ectopic expression of *STF* in switchgrass, rice and *Brachypodium* leads to significant increase in plant size primarily through promoting cell proliferation.

**Fig 3:**
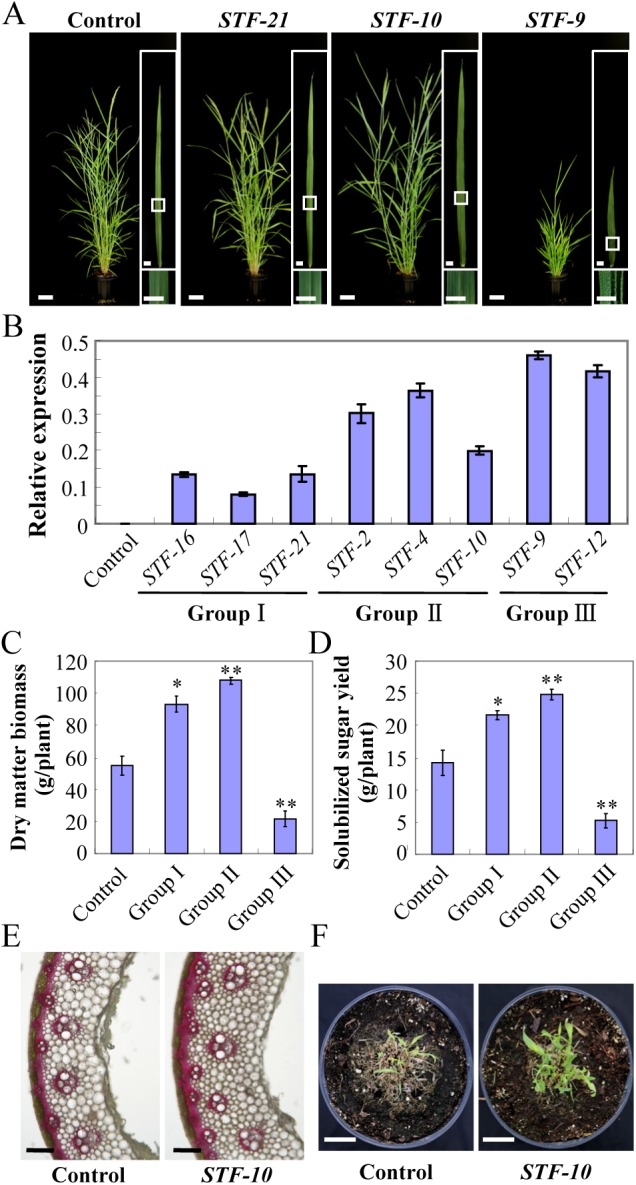
*STF* overexpression in switchgrass improves biomass yield and release of solubilized sugars. (A) Morphology of switchgrass plants overexpressing different levels of *STF* at flowering. One representative from each group was shown. Bars = 10 cm for plants, 1cm for leaves. (B) Transcript abundance of *STF* in transgenic plants revealed by qRT-PCR. *UBI*::*GUS-1* switchgrass plant was used as the control. Bars represent means ± SE of three technical replicates. (C) Comparison of postharvest dry weights of total above-ground biomass of three control (*UBI*::*GUS* plants) and three classes of *STF* overexpressors shown in (B) at maturity. Bars represent means ± SE (n = 3 independent plants for Control, Group Ⅰ, Ⅱ, and 2 plants for group Ⅲ), the asterisks indicate significant differences (*p<0.05, **p < 0.01, Student t-test). (D) Solubilized sugar yield of transgenic switchgrass plants compared to control (*UBI*::*GUS* plants) shown in (B). Bars represent means ± SE (n = 3 plants for Control, Group Ⅰ and Ⅱ, 2 plants for group Ⅲ), the asterisks indicate significant differences (*p<0.05, **p < 0.01, Student t-test). (E) Phloroglucinol-HCl staining of lignin in the internode Ⅲ of the control (*UBI*::*GUS-1*) and Group Ⅱ *STF-10*. Bars = 100 μm. (F) Recovery and growth establishment after shoot harvest. The *STF* transgenic switchgrass (Group Ⅱ *STF-10*) displays better recovery after cut back compared to the control (*UBI*::*GUS-1*). Plants shown were 2 weeks old after cutting. Bars = 5 cm.

To evaluate the impact of *STF* on biomass production, we measured agricultural traits in the bioenergy crop switchgrass including leaf blade length and width, plant height, internode diameter, tiller number and flowering time (S1 Table), which divided the transgenic switchgrass lines into three categories: Group Ⅰ, Group Ⅱ and Group Ⅲ. Representative lines for each group were shown in Fig 3A. Among these, the transgenic lines with high *STF* transcript levels (Group Ⅲ) displayed severe morphological alteration including twisted and curled leaf blade, reduced internode and plant height and delayed flowering. The low and moderate *STF* expressing transgenic lines, Group Ⅰ and Group Ⅱ, respectively, on the other hand, exhibited normal or even slightly enhanced plant height resulting in an overall improved plant stature (Fig 3B). *STF* transgenic rice and *Brachypodium* lines also showed a similar dosage-dependent effect on overall growth and development (S1 and S2 Figs).

To quantitatively determine these improvements, we evaluated total above ground dry biomass yield after maturity. We evaluated three independent transgenic lines in Group Ⅰ (STF-16, 17, and 21) and Group Ⅱ (STF-2, 4, and 10), and two independent lines in Group Ⅲ (STF-9 and 12) using three independent *UBI*::*GUS* transformed controls. The average dry weight of Group Ⅰ and Group Ⅱ transgenic switchgrass had a 1.68 and 1.95 fold increase, respectively, in total biomass compared with the controls, whereas strong *STF* expression in the group Ⅲ transgenic switchgrass led to reduced biomass production lower than controls due to stunted growth (Fig 3C). Further enzymatic hydrolysis of the dry biomass without pretreatment indicated that, excepting the Group Ⅲ transgenic lines, the total amount of solubilized sugar yield of Group Ⅰ and Group Ⅱ had increased by over 1.53 and 1.75-fold per plant, respectively (Fig 3D). This is a significant improvement in the feedstock properties of switchgrass for cellulosic ethanol production because more solubilized sugar implies more ethanol without pretreatment.

Lignin negatively impacts biomass recalcitrance and reduces bioconversion to ethanol. Composition analysis of dried *STF* transgenic switchgrass was carried out to further assess lignin content and its amenability to biofuel production. Measurement of acid detergent lignin content (ADL) and Phloroglucinol-HCl staining showed no obvious difference in lignin deposition pattern or ADL content in Group Ⅰ and Group Ⅱ transgenic switchgrass compared with the controls, whereas the lignin content in Group Ⅲ transgenic plants was slightly decreased (Fig 3E and S2 Table). Cellulose and hemicellulose analysis also indicated no difference in Group Ⅰ and Group Ⅱ, but slightly decreased in Group Ⅲ (S2 Table). These biochemical analyses indicated that overexpression of *STF* could significantly increase biomass yield and sugar release without altering relative cell wall components, demonstrating a great potential of *STF* to developing superior switchgrass varieties for biofuel production. Moreover, we observed that the Group Ⅰ and Group Ⅱ transgenic plants displayed better regenerative capacity in forming new leaves after harvest cutting (Fig 3F), suggesting that weak and moderate level of *STF* expression is conducive to facilitated switchgrass growth even at early developmental stages.

**Fig 4:**
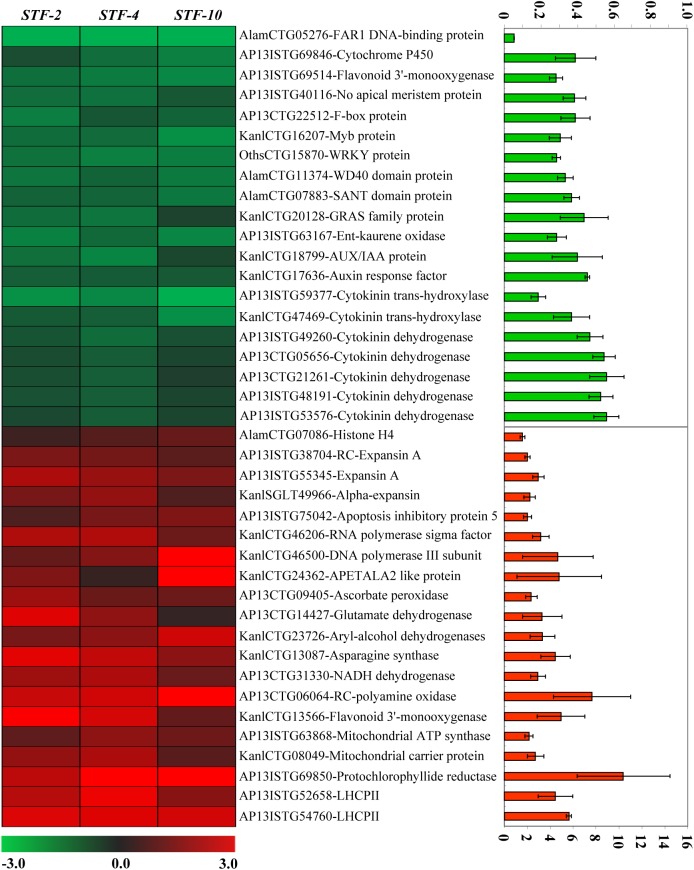
A heat map showing some representatives of differentially expressed genes from *STF* transgenic switchgrass microarray analysis. Selected genes related to cell division, phytohormones, metabolic processes and transcription factors were shown. Profiling analysis was performed in 6–8 cm newly generated tillers, approximately 3 weeks after cutting, of three independent *STF* overexpressing Group Ⅱ switchgrass lines and three independent *UBI*::*GUS* control lines. The log ratio values (vs control average) were used to construct the heat map with the scale bar at the bottom showing expression intensities. Red colors represent upregulated genes whereas green colors represent downregulated genes. The column on the right shows the fold changes of the selected genes in the *STF* overexpressing switchgrass plants compared to the controls based on the microarray data. Bars represent means ± SE (n = 3).

To gain insight into the mechanistic effects of *STF* on cell proliferation and overall plant development, we performed transcript profiling analysis using the switchgrass Affymetrix GeneChip and compared gene expression between three independent *STF* overexpressing Group Ⅱ plants and three independent controls transformed with *GUS*. Profiling analysis was performed in 6–8 cm newly generated tillers approximately 3 weeks after cutting. A total of 886 probes were significantly altered with a 2-fold or more difference compared to controls, in which 665 probes were downregulated (S3 Table) and 221 probes were upregulated (S4 Table), consistent with the primarily transcriptional repression function of *STF* in its native host. Gene Ontology assignments indicated that a wide range of functional groups were represented in both the upregulated and downregulated genes (S3 Fig). In agreement with the histological observation, this analysis identified genes that are known to be involved in cell proliferation such as *Histone H4* and *Expansin* as upregulated in *STF* transgenic plants (Fig 4), which were confirmed by qRT-PCR (S4 Fig). The microarray data analysis also revealed that putative *cytokinin oxidase/dehydrogenase* (*CKX*) genes were downregulated in *STF* transformants (Fig 4, Table 1). Cytokinin oxidases/dehydrogenases catalyze the irreversible degradation of cytokinins and play important roles in maintaining cytokinin homeostasis. The phytohormone cytokinin (CK) affects many aspects of plant developmental programs, including a prominent role in the regulation of cell proliferation, plant growth and determination of organ size [41]. It is plausible that increasing the CK levels by reducing expression of *CKXs* could result in enhanced biomass and grain production as well as increased plant stature [42, 43]. However, several genes involved in auxin signaling/response were also altered in expression (Table 1). The phenotypes of *STF* transgenic lines in the three grass species and the microarray data prompted us to think that the effect of *STF* may, at least in part, be connected to cytokinin levels.

**Table 1:** Hormone-Associated Genes Differentially Expressed in *STF* Overexpressing Switchgrass

Probe sets	Putative annotation	Fold change^a^	P-value^b^	Putative function^c^
AP13CTG02927_at	3-β hydroxysteroid dehydrogenase	0.075	0.00228	Steroid hormones biosynthesis
AP13ITG51564_s_at	3-β hydroxysteroid dehydrogenase	0.088	0.00072	Steroid hormones biosynthesis
AP13ITG59377_s_at	Cytokinin trans-hydroxylase	0.112	0.01188	Cytokinin biosynthesis
AP13ITG69514_s_at	Flavonoid 3'-monooxygenase	0.282	0.02789	Auxin biosynthesis
AP13ITG67646_at	Glutathione s-transferase	0.324	0.00761	Auxin and cytokinin response
KanlowCTG47469_s_at	Cytokinin trans-hydroxylase	0.366	0.01454	Cytokinin biosynthesis
KanlowCTG18799_at	AUX/IAA	0.397	0.01400	Auxin signaling
KanlowCTG17636_x_at	Auxin response factor	0.452	0.00033	Auxin signaling
AP13ITG49260_s_at	Cytokinin dehydrogenase	0.466	0.00279	Cytokinin degradation
AP13ITG48191_s_at	Cytokinin dehydrogenase	0.525	0.00109	Cytokinin degradation
AP13ITG64496_at	Glutathione s-transferase	2.392	0.00712	Auxin and cytokinin response
AP13ITG66839_s_at	ABA induced protein	2.749	0.00380	ABA response
AP13ITG65716_at	Horseradish peroxidase	3.817	0.05530	Auxin catabolism

^a^Relative abundance of transcript in *STF* overexpression/control (*UBI*::*GUS*) switchgrass plants.

^b^P-value calculated as described in materials and methods.

^c^Category of predicted gene function.

**Fig 5:**
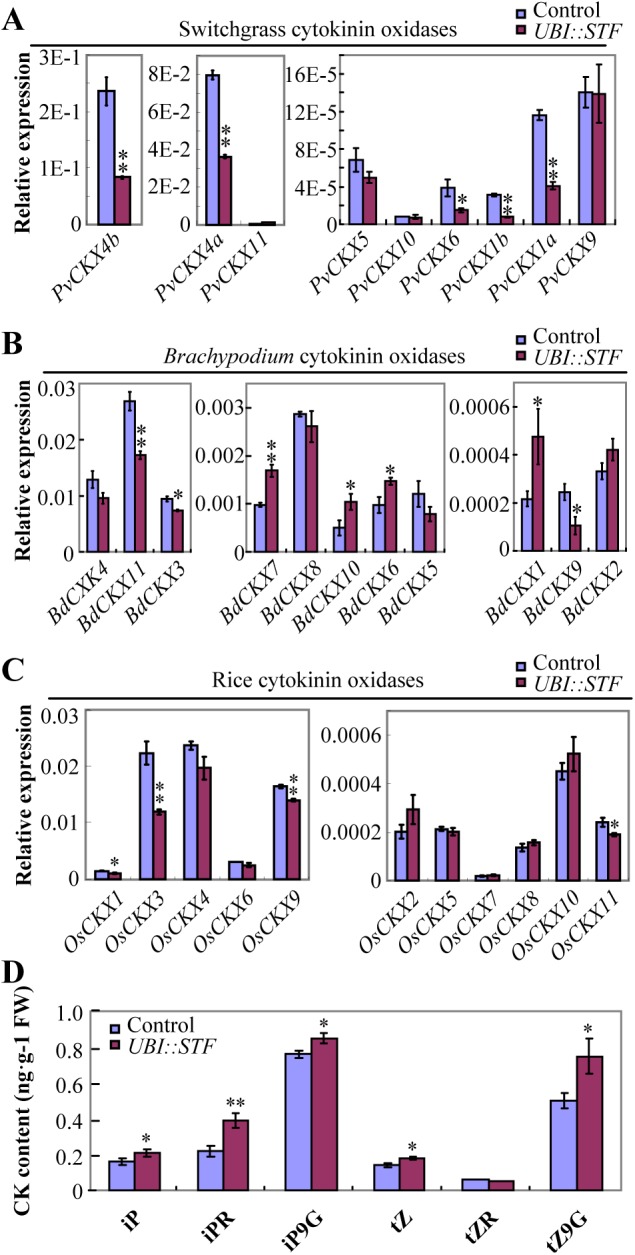
Transcript abundance of *CKX* genes and cytokinin (CK) content in *STF* overexpressing transgenic grasses. (A) Transcript levels of *CKX* genes in the 6–8 cm newly generated tillers, approximately 3 weeks after cutting, of control (*UBI*::*GUS*) and *STF* overexpressing switchgrass. Bars represent means ± SE of three technical replicates and two biological replicates. (B) Transcript levels of *CKX* genes in 4 weeks old seedling of control (*UBI*::*GUS*) and *STF* overexpressing *Brachypodium*. Bars represent means ± SE of three technical replicates and two biological replicates. (C) Transcript levels of *CKX* genes in 2 weeks old seedlings of control (*UBI*::*GFP*) and *STF* overexpressing rice. Bars represent means ± SE of three technical replicates and two biological replicates. (D) Comparison of CK content in the top 2 leaves of 2 months old wild type and *STF* overexpressing rice. iP, isopentenyladenine; iPR, iP riboside; iP9G, iP 9-glucoside; tZ, zeatin; tZR, zeatin riboside; tZ9G, zeatin 9-glucoside. Bars represent means with SE of three technical replicates from three independent wild type and *UBI*::*STF* plants. The asterisks indicate significant differences (* p<0.05, ** p < 0.01, Student t-test). FW, fresh weight.

To confirm that overexpression of *STF* in switchgrass, *Brachypodium* and rice reduced *CKX* gene expression, we isolated 9 CKX family members from switchgrass and 11 members from each of *Brachypodium* and rice and examined their transcript levels by qRT-PCR. We found that the expression levels of 5 out of 9 PvCKX family members were considerably reduced in *STF* overexpressing switchgrass (Fig 5A). Similarly, the transcript levels of three BdCKX family members in *Brachypodium* and four OsCKX family members in rice were also found to be significantly reduced in *STF* transgenic lines compared to the controls (Fig 5B and 5C), indicating that the downregulation of *CKXs* in transgenic switchgrass microarray was not an isolated event. The *CKXs* that are repressed by *STF* across the three species are phylogenetically close to each other except *PvCKX6*, which appears to have no partners acting in the same way in rice or *Brachypodium* (S5 Fig). Since CKX enzymes are responsible for cytokinin degradation, we directly measured active and transiently inactive cytokinin levels in the *STF* overexpressing rice plants, for which optimized methods have been established [44]. We found that the amount of 5 of the 6 CK species measured, iP, isopentenyladenine; iPR, iP riboside; iP9G, iP 9-glucoside; tZ, zeatin; tZ9G, zeatin 9-glucoside, were significantly increased in *STF* overexpressing rice than the control plants (Fig 5D, S5 Table), suggesting that the repression of *CKX*s has a biological significance in promoting cytokinin levels. These data are consistent with the cell proliferation promotion activity of *STF* and suggest that the enhanced cell proliferation in *STF* transformants could in part be caused by elevated CK levels through repressing the expression of CKX family CK degrading enzymes, providing a novel mechanistic insight for *STF* molecular function.

**Fig 6:**
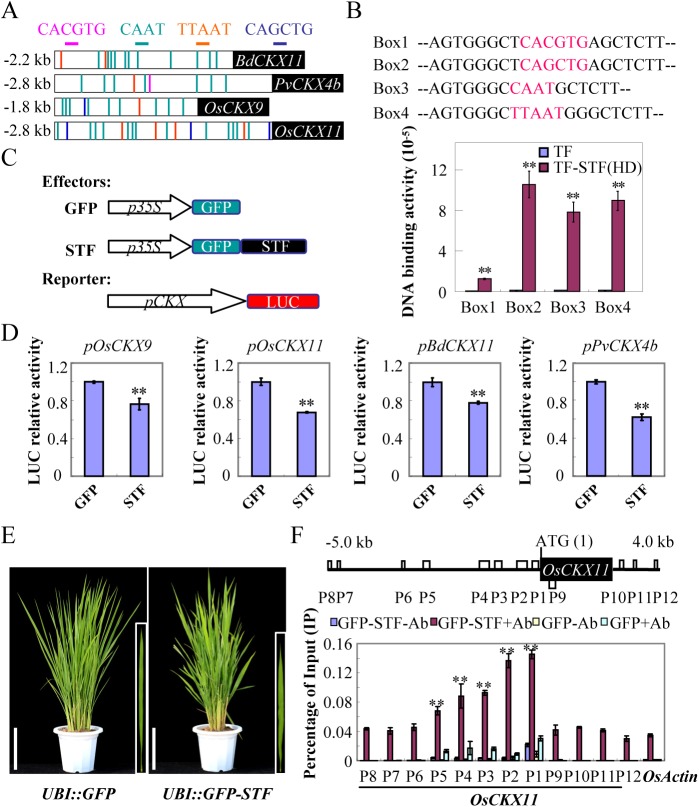
STF directly binds and represses the expression of several *CKX* genes *in vitro* and *in vivo*. (A) Schematic presentation of putative STF binding sites in *CKX* promoters. (B) DNA binding assay corresponding to the putative binding sites of STF. DNA fragments bound to His-TF-STF(HD) fusion protein or His-TF control were quantified by qRT-PCR after elution. Bars represent means ± SE of three technical replicates and two biological replicates. The asterisks indicate significant differences (** p < 0.01, Student t-test). (C) Effector and reporter constructs used in transient dual luciferase assay. (D) Dual luciferase assay showing repression of rice, *Brachypodium* and switchgrass *CKXs* by the STF effector construct compared to the GFP control effector construct. Bars represent means ± SE of three technical replicates, and two biological replicates. The asterisks indicate significant differences (** p < 0.01, Student t-test). (E) Phenotype of *UBI*::*GFP-STF* T1 transgenic rice plant and *UBI*::*GFP* control from which 10-day old T2 seedlings were taken for the ChIP experiment. Bars = 10 cm. (F) ChIP assay showing the association of STF with several regions in the promoter of *OsCKX11*. The boxes indicate the position of the specific binding sites (P1-P5) and non-specific sites used as control (P6-P12). Bars represent means ± SE of three technical replicates and two biological replicates. The asterisks indicate significant enrichment (** p < 0.01, Student t-test) compared to the *OsActin* control.

Owing to the fact that STF acts primarily as a transcriptional repressor [36, 38] and specific *CKXs* are repressed in *STF* overexpressing switchgrass, *Brachypodium* and rice transgenic lines, we hypothesized that STF may directly repress the CKX family genes to promote CK activity. To test this hypothesis, we performed three complimentary experiments. First, we tested direct DNA binding of STF to *CKX* promoters *in vitro* using DNA binding assay. WOX family proteins have been reported to bind the G-box, the known TAAT motif as well as consensus sequences like CAAT and TTAA [31, 45]. Sequence analysis revealed that these binding sequences were present in the proximal promoter regions of the downregulated CKX family members like *OsCKX9* and *OsCKX11* in rice, *BdCKX11* in *Brachypodium* and *PvCKX4b* in switchgrass (Fig 6A). We tested whether the STF protein can directly bind to DNA fragments containing these sequences *in vitro*. The STF homeodomain region was fused to a Trigger Factor (TF), a molecular chaperone protein that improves protein solubility [46] for expression in *E*.*coli* and purified using Profinity IMAC Ni-Charged Resin (BIO-RAD). Our results showed that the recombinant TF fused STF homeodomain protein was able to bind the fragments containing the conserved G-box, TAAT motif and CAAT and TTAA sequences, while binding was not detected by the control TF protein alone (Fig 6B), suggesting that the STF repression of *CKXs* is mediated by direct binding to their specific promoter sequences.

Second, using a well-established transient dual luciferase assay system in rice leaf protoplasts, we found that coexpression of the STF effector protein and the luciferase reporter constructs driven by the 1.8–2.8 kb promoters of *OsCKX9* and *OsCKX11* (Fig 6C), both downregulated in *STF* overexpressing rice, resulted in a significant reduction of the luminescence intensity compared with the GFP control effector protein (Fig 6D). Similarly, coexpression of the STF effector with the switchgrass *pPvCKX4b* and *Brachypodium pBdCKX11* reporter constructs significantly reduced luminescence in the rice protoplast luciferase assay (Fig 6D), indicating that STF recognizes these *CKX* promoters in protoplasts and represses their activity.

Third, we tested direct *in vivo* binding of STF protein to *OsCKX* promoters by Chromatin Immunoprecipitation (ChIP) assay. Using *UBI*::*GFP-STF* transgenic rice and anti-GFP antibody, we found that the promoter regions of *OsCKX9* and *OsCKX11* were enriched in the GFP-STF chromatin (Fig 6E and 6F and S6 Fig). We tested five potential binding sites (P1-P5) within 2.8 kb region upstream of the translational start site, three non-specific regions (P6-P8) further upstream, one in the coding region (P9) and three non-specific regions downstream of the translation stop site (P10-P12) of *OsCKX11* (Fig 6F). We found that the P1-P5 putative binding regions were significantly enriched compared to the non-specific regions (P6-P12) or the background signal in the *OsActin* control (Fig 6F), indicating that STF directly binds to multiple regions on *CKX* promoters, consistent with the DNA binding and dual luciferase assay results. These results together highlight a mechanism by which STF may act to modulate cytokinin levels in leaf development. Our data also demonstrate that ectopically expressing the *Medicago WOX* gene *STF* in switchgrass promotes leaf blade outgrowth and biomass accumulation with increased solubilized sugars, suggesting a potential in biomass feedstock improvement.

## Discussion

The plant-specific WOX family transcription factors are master regulators of plant growth and development including shoot and root apical meristems, lateral organs, organ size and vasculature [28, 47–53]. The *Medicago WOX* gene *STF* and its orthologues are key regulators of leaf blade outgrowth in the medial-lateral direction and floral organ fusion in several eudicot species [33–35, 54]. Here we reported that ectopic expression of *STF* in switchgrass, *Brachypodium* and rice universally promotes cell proliferation in vegetative organs leading to wider leaf blades, thicker stems and overall significant increase in total biomass yield. Lignocellulosic biomass has a potential to displace a significant portion of gasoline as a renewable energy source [1, 6, 55], and switchgrass is one of the dedicated bioenergy crops in the United States for lignocellulosic biofuel production [7, 56]. Although a 1.3 billion ton biomass production capacity per annum was projected in the US alone, this estimation takes into account that current biomass production efficiency per unit of land would be at least doubled including in marginal lands that are not in current crop production systems [2], indicating that biomass yield is still a major challenge for sustainable biofuel production. Another major hurdle is bioconversion efficiency imposed by the cell wall lignin polymer. Lignin, a phenolic polymer, is a major component of most plant cell walls which is recalcitrant to saccharification through enzymatic digestion [4, 5]. Lignin forms complex cross-links with two other major cell wall polymers, cellulose and hemicellulose, making these components inaccessible to saccharification without pretreatment with strong acids. This indicates that deconstruction of plant cell walls is a significant challenge and lignin content is an important biomass feedstock trait determining bioconversion efficiency. In fact, reducing lignin content by genetic engineering for feedstock quality improvement is currently a major undertaking in several laboratories. Here we demonstrate that it is possible to contribute to both biomass yield and sugar release in switchgrass and other grasses using the leaf development master regulator *STF*. Switchgrass Alamo plants overexpressing *STF* showed approximately a 2-fold increase in above ground total dry weight production and approximately 1.8-fold increase in the release of solubilized sugars (Fig 3), improving biomass feedstock properties without necessarily altering the relative composition of cell wall polymers. Similar successes have been reported in switchgrass using maize *Corngrass1* miRNA [10] and rice miRNA156 [9] resulting in improved saccharification efficiency by altering developmental phase changes. Overexpression of a switchgrass *ERF* gene *PvERF001* was also recently reported to increase biomass yield [8], but to our knowledge, this is the first report showing significant improvement in biomass yield and sugar release without pretreatment using a WOX transcription factor in switchgrass, providing a new tool for genetic modification of grasses.

*STF* and its orthologues are unique among the leaf blade regulators in the sense that they are expressed at the adaxial-abaxial juxtaposition [33–35, 54] unlike the well-studied polarity factors that are axial-specific. In this middle region at the leaf margin and middle mesophyll, *STF* and *Arabidopsis WOX1* promote lateral expansion of the leaf blade by activating cell proliferation [33, 35] analogous to *WUS* in the SAM [50] and *WOX5* in the RAM [48, 57]. *STF* is clearly shown to affect free auxin and ABA levels by direct measurements but has also been proposed to act as a master switch affecting several developmental programs probably through regulating multiple hormone homeostasis including auxin, cytokinin, and metabolic sugars based on transcriptomic, transgenic and metabolomics data analyses [33, 39]. But the detailed mechanism is unknown. STF physically interacts with the transcriptional co-repressor MtTPL and primarily acts as a transcriptional repressor for its cell proliferation activity in leaf blade outgrowth [36–38]. Since *Arabidopsis* TPL is known to modulate auxin signaling via interaction with repressive auxin response factors (ARFs) [58], it could be assumed that at least the STF-TPL complex may recruit ARFs to explain the observed effects of STF on auxin levels. However, *ARF* gene expression was also altered in the *Medicago stf* mutant microarray data [33], in the current *STF* transgenic switchgrass microarray, as well as in *Arabidopsis wox1/prs* double and petunia *maw* mutants quantified by qRT-PCR [34], suggesting a potential for *STF* direct effects on auxin signaling/homeostasis, although such direct effects are yet to be shown mechanistically.

**Fig 7:**
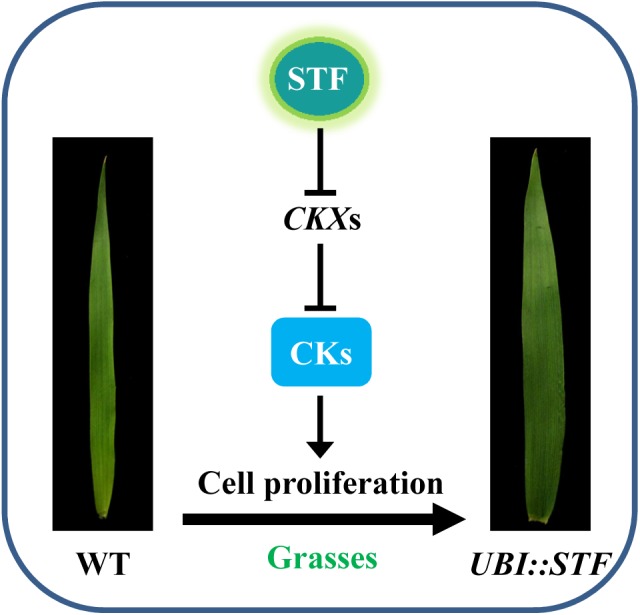
A simplified hypothetical model depicting *STF*-mediated leaf blade expansion in transgenic grasses. STF transcriptionally represses the expression of cytokinin (CK) degrading cytokinin oxidases/dehydrogenases (*CKX*s) by directly binding to their promoters. This presumably increases active CK levels in leaf tissues which might contribute to enhanced cell proliferation leading to broader leaf blades.

Here we demonstrate that STF can directly bind to multiple regions on the promoter of cytokinin oxidases/dehydrogenases (*CKXs*) *in vitro* and *in vivo*, and represses their transcription in transgenic switchgrass, *Brachypodium* and rice (Figs 5 and 6), providing a novel mechanism to control local cytokinin homeostasis. *CKXs* are induced by cytokinins in plant tissues and catalyze the degradation of active cytokinins in a feedback inhibition to maintain cytokinin homeostasis [59]. This suggests that STF improves active cytokinin pools by preventing cytokinin degradation through repressing *CKX* genes (Fig 7). Indeed, direct measurement of active cytokinins and directly convertible cytokinin conjugates confirmed that 5 of the 6 cytokinin species analyzed were significantly increased in *STF* overexpressing transgenic rice lines. Since cytokinins are major regulators of cell proliferation [60], this finding is consistent with the STF’s role in the promotion of cell proliferation by transcriptional repression activity in leaf blade outgrowth and total biomass accumulation in the native *Medicago* and transgenic grasses. Activation of cytokinin signaling by *WOX* genes has been established for *WUS* in *Arabidopsis* SAM maintenance through repression of A-type *ARR*s including *ARR5*, *ARR6*, *ARR7* and *ARR15*, which are negative regulators of cytokinin signaling [49, 61]. *WOX9/STIP* is also reported to act downstream of cytokinin sensing in the *Arabidopsis* SAM and proposed to activate the A-type *ARR*s [62]. Rice *OsWOX4*, that performs an equivalent function to *Arabidopsis WUS* in shoot meristem maintenance in rice, has also been reported to mimic cytokinin application in inducing calli in transgenic rice [63] although the mechanism is not known. Activation of cytokinin activity by repressing *CKX*s may thus be yet another mechanism to control cytokinin response, which may also have important implications in SAM maintenance. We checked the effect of *STF* on *CKX*s in *Medicago truncatula*, where *STF* is native. In the *stf* mutant microarray, one of only two genes included in the chip (*Medtr4g044110*) was modestly upregulated at 1.32 and 1.47 times, for 2 different probe sets, in the mutant compared to wild type, the other gene was unchanged at 1.08 [33]. We were able to identify seven definitive *CKX*-like genes in the annotated version 3.5 of the *M*. *truncatula* genome. We tested the expression of all of them by semi quantitative PCR in the unexpanded young leaves of wild type R108 and two *stf* mutant alleles, *stf-1* and *stf-2*. Our preliminary results showed that one of them (*Medtr3g036100*) was significantly induced in the mutants, and two others, *Medtr4g044110* and *Medtr2g039410*, were slightly induced, while the other four were basically unchanged (S7 Fig). The *Medtr4g044110* weak induction in the mutants is consistent with the *stf* microarray data. This preliminary observation suggests that the effect on cytokinin activation may be part of the *STF* function in eudicots. In fact, the first true leaf-like structure with apparent petiole and blade outline was obtained in *Nicotiana sylvestris lam1* mutants only after application of auxin and cytokinin together to the shoot apex [33], suggesting that both auxin and cytokinin signaling pathways and/or their crosstalk could be components of the *STF* function in leaf blade outgrowth.

Nevertheless, *STF* homologues are not found in monocots [33, 34, 38, 64, 65] and the biological significance of the cytokinin mechanistic model is yet to be tested by the endogenous grass *WOX* gene(s). In monocots, the WOX3 family is proposed to perform an equivalent function [39]. At least in maize and rice, where information is available, the function of *STF/WOX1* appears to be met by the WOX3 family members *NS1* and *NS2* in maize [27], and *NAL2* and *NAL3* in rice [29, 30]. In eudicots, one *WOX3/PRS* and at least one *STF/WOX1* genes are present but monocots have at least two *WOX3/NS* genes instead. *Arabidopsis PRS* is required for lateral axis-dependent development of flowers, lateral sepals, lateral stamens and leaf stipules [27, 28, 66], but *prs* mutants do not display narrow leaf blades although *PRS* is found to redundantly function with *WOX1* in leaf blade outgrowth [34, 35]. In *M*. *truncatula* and probably other eudicots, the *WOX3/LFL* role is restricted to flower development [52]. It is likely that *WOX1* and *WOX3* have separate roles in most eudicots, other than *Arabidopsis*, and leaf blade development in the medial-lateral axis is governed by the STF/WOX1 family, while redundant WOX3 family genes fulfill this role in monocots. Thus, it is not surprising if some of the mechanisms are conserved between *STF/WOX1* and *WOX3/NS* functions. The actual mechanism of *NS* and *NAL* function is unclear but the involvement of hormone signaling, especially auxin, has been predicted from transcriptome analysis [29, 32] and OsWOX3A has been reported to act in GA homeostasis [31]. It would be interesting to see if the *ns1/2* and *nal2/3* mutants have altered cytokinin activity or *CKX*s expression levels, and also complement these mutants with *STF/WOX1* to understand the extent of mechanistic conservation between monocots and eudicots in lateral outgrowth of the leaf blade.

Our data suggest that promotion of cytokinin activity by direct repression of cytokinin degradation may be a general mechanism for the action of repressive *WOX* genes that are involved in cell proliferation during meristem maintenance and lateral organ development including *WUS*, *NAL* and *NS* genes. Further experiments are needed to confirm the validity of this hypothesis. Taken together, our results provide a powerful tool for genetic modification of biomass yield and sugar release in perennial and annual grasses, and uncover a novel mechanistic insight directly connecting cytokinin activity and cell proliferation by repressive *WOX* genes that have far reaching consequences in regulating plant vegetative and reproductive developmental programs.

## Materials and methods

### Plant materials and growth conditions

*Brachypodium distachyon* inbred line *Bd21-3*, *Oryza sativa* varieties *Nipponbare* and the lowland-type switchgrass cultivar Alamo were used in this study. *Brachypodium* and switchgrass plants were grown in the greenhouse under a 26°C/16-h (day) and 23°C/8-h (night) photoperiods with lighting supplied by parabolic aluminized reflector lamps (average 390 μE⁄m^2^⁄S1). The rice (*Oryza sativa* L.) plants were cultivated in experiment field and green house in Beijing.

### Vector construction and plant transformation

The Maize Ubiquitin promoter from pTCK303 [67] was cloned into pMDC32 Gateway vector substituting *2×35S* promoter to generate pMDC32-pUBI destination vector by *Hin*d Ⅲ and *Kpn* Ⅰ, and *STF*, *GFP* and *GUS* were cloned into pMDC32-pUBI destination vector by using the Gateway system (Invitrogen). To generate the *UBI*::*GFP-STF* vector, *GFP* and *STF* were cloned separately, with 18 bp overlapping sequence between 3’*GFP* and 5’*STF* to acquire the *GFP-STF* sequence cloned into pMDC32-pUBI destination vector by using the Gateway system (Invitrogen). Constructs were introduced into *Agrobacterium tumefaciens* by electroporation or the freezing transformation method. *A*. *tumefaciences* strain *AGL1* was used for *Brachypodium*, switchgrass and rice transformation as previously described [9, 68, 69].

### RNA extraction and quantitative RT-PCR

Total RNA was extracted from 6–8 cm newly generated tillers approximately three weeks after cutting (for switchgrass Microarray and qRT-PCR analysis), 2 weeks old seedlings (for rice qRT-PCR analysis) and 1 month seedling shoots (for *Brachypodium* qRT-PCR analysis) of *UBI*::*STF*, *UBI*::*GUS* and *UBI*::*GFP* transgenic plants by using TRIzol Reagent (Invitrogen). cDNA was generated by reverse transcription with SuperScript Ⅲ (Invitrogen). Quantitative RT-PCR was performed as previously described [36], with at least 2 biological and 3 technical replicates for both samples and controls. The *CKX* gene ID and primers used were listed in S6 and S7 Tables.

### Histological analysis

Tissue fixation and embedding were performed as described [69]. The tissues were sliced into 10 μm sections with a Leica RM2145 microtome, affixed to microscope slides, and stained with toluidine blue. Cross sections of the flag leaves and internode Ⅱ (for rice and *Brachpodium*) and internode Ⅲ (for switchgrass) of the controls and *STF* transformants were stained with phloroglucinol-HCl reagent as previously described [70]. Images were taken under an Olympus BX-51 compound microscope.

### Microarray analysis

6–8 cm newly generated tillers, approximately 3 weeks after cutting, of three independent Group Ⅱ *UBI*::*STF* transgenic lines (*STF-2*, *4*, and *10*) and three independent *UBI*::*GUS* lines were used for total RNA extraction and microarray experiment. The Microarray analysis of transgenic switchgrass plants were performed as previously described [9]. Data analysis of differentially expressed probe sets on the chip was performed by associative analysis as described [71].

### Quantification of cytokinins

Extraction and determination of CKs from the top two leaves of three *UBI*::*STF* transgenic rice lines and wild type at vegetative stage (two months old after planting) were performed by using a polymer monolith microextraction/hydrophilic interaction chromatography/electrospray ionization tandem mass spectrometry method as described [44].

### Biofuel property analysis

Total above-ground shoot tissues at flowering stage were harvested for the dry biomass yield analysis and further biofuel property evaluation. Lignin analysis and enzymatic saccharification were performed as previously described [9].

### DNA binding assay

The *STF* cDNA corresponding to the N-terminal and homeodomain (HD) regions with amino acids 1 to 226 was cloned into the *E*. *coli* expression vector pCOLD-TF with His tag (Takara, TF indicates Trigger Factor, a molecular chaperone protein of original nuclear pullulan increasing the protein solubility) using *Eco*R Ⅰ and *Bam*H Ⅰ restriction enzymes. Expression of pCOLD-TF, and pCOLD-TF-STF(HD) in BL21 cells was induced with 0.2 mM isopropyl-1-thio-D-galactopyranoside at 18°C for 16 h. Fusion protein was purified using Profinity IMAC Ni-Charged Resin (BIO-RAD) according to the manufacturer’s protocol and quantified by the Bio-Rad protein assay reagent. DNA binding assay was performed as previous described [72]. The putative STF binding fragments were incubated with purified His-TF alone and with the His-TF-STF(HD) fusion protein, and the DNA binding activity (protein bound DNA) was determined by qRT-PCR after washing and elution. Primers used were listed in S7 Table.

### Transient lucifarese assay

The coding sequences of GFP-STF and GFP were cloned into p2GW7 using the Gateway system (Invitrogen) to yield effector plasmids. For the reporter plasmid, a mini *35S* promoter [73] with *Bam*H Ⅰ was inserted into the pGreen Ⅱ-0800-Luc vector by exonuclease Ⅲ, to generate the destination vector pGreen Ⅱ-0800-p35S mini-Luc, and the promoter of *OsCKX9*, *OsCKX11*, *BdCKX11 and PvCKX4b* were cloned into pGreen Ⅱ-0800-p35S mini-Luc by restriction digestion to generate the reporter plasmid. Primers used were listed in S7 Table. Transient expression assays were performed in rice protoplasts as previously described [36, 74]. For each transformation, 5 μg of reporter plasmid and 5 μg of effector plasmid were used. Luciferase activities were detected by Dual-Luciferase Reporter Assay System (Promega) as previously described [75].

### ChIP assay

The *UBI*::*GFP* and *UBI*::*GFP-STF* transgenic rice lines were used for ChIP assay according to the method described previously [76] with some modifications. Briefly, 1 g tissue of 10-day-old T2 seedlings per sample was harvested from plants grown in greenhouse. Samples were cross-linked with 1% (v/v) formaldehyde under vacuum for 10 min, quenched with Gly (0.2 M) for 5 min, and then ground to powder in liquid nitrogen. The chromatin complexes were isolated, sonicated and then incubated with polyclonal anti-GFP antibodies (Abcam, AB290). The precipitated DNA was recovered and used as a template for qRT-PCR analysis. The input DNA and no antibody–precipitated DNA were used as positive and negative controls, respectively. The primers used for the ChIP assays were described in S7 Table.

## Supporting information

S1 Fig Morphological characterization and molecular analysis of *STF* overexpression in rice. (A) Phenotypes of three classes of *STF* overexpressing rice lines. The control was transformed in the same way with *UBI*::*GFP*. Bars = 10 cm in upper row, 2 cm in middle row and 2 mm in lower row. (B) Transcript abundance of *STF* in transgenic plants revealed by qRT-PCR. Bars represent means ± SE of three technical replicates.
(TIF)

S2 Fig Morphological characterization of *STF* overexpressing *Brachypodium*. (A) Phenotypes of *STF* overexpressing *Brachypodium*. *UBI*::*GUS* transformed *Brachypodium* was used as the control. Bars = 10 cm. (B) Transcript abundance of *STF* in transgenic plants revealed by qRT-PCR. Bars represent means ± SE of three technical replicates. (C) Comparison of seed size between *STF* transgenic and control (*UBI*::*GUS*) plants. Bar = 1 mm.
(TIF)

S3 Fig Predicted functions of differentially expressed genes in *STF* overexpressing switchgrass. A pie chart representing the distribution of functional classifications of down-regulated (A) and up-regulated (B) probes based on the Gene Ontology Assignments.
(TIF)

S4 Fig Expression of *Histone H4* and *Expansin* genes in *STF* overexpressing switchgrass. Transcript levels of genes encoding putative *Histone H4*, *Expansin A* and *Alpha-expansin* in *STF* transgenic lines revealed by qRT-PCR. *UBI*::*GUS* expressing switchgrass plants were used as controls. Bars represent means ± SE of three technical replicates and two biological replicates. The asterisks indicate significant differences (** means p < 0.01, Student t-test).
(TIF)

S5 Fig Phylogenetic analysis of Cytokinin Oxidases in *Brachypodium*, rice and switchgrass. Full-length amino acid sequences were aligned using Clustal W and the tree was constructed using MEGA4 with 1000 replicates. Species: Os, *Oryza* *sativa;* Bd, *Brachypodium distachyon;* Pv, *Panicum virgatum*. The green dots highlight the downregulated *CKXs* in *STF* overexpressing grasses.
(TIF)

S6 Fig STF directly binds to the promoter of *OsCKX9 in vivo*. (A) Schematic representation of the regions of *OsCKX9* tested by ChIP experiments. P1-P5 are specific STF binding sites, while P6-P9 are non-specific sites used as control. (B) ChIP assay showing the association of STF with several regions in the promoter of *OsCKX9* (P1-P5) compared to background signal (P6-P9) or the *OsActin* negative control. Bars represent means ± SE of three technical replicates and two biological replicates. The asterisks indicate significant differences (** p < 0.01, Student t-test).
(TIF)

S7 Fig *Medicago CKX* expression in *stf* mutants. Semi quantitative RT-PCR showing the expression of seven *CKX* genes in the leaves of four weeks old *M*. *truncatula stf* mutants compared to wild type. Numbers on the right show the number of PCR cycles used. * represents a gene that showed weak induction in *stf* microarray.
(TIF)

S1 Table Morphological characterization of *STF* transgenic switchgrass plants. Plant height and tiller number of *STF* transgenic and control switchgrass plants were measured after 4-months of growth in the greenhouse. 7 tillers were used to measure internode length and diameter (internode Ⅱ), leaf blade length and width were measured in the 3rd leaf for each plant. The control represents the average of three independent *UBI*::*GUS* transgenic plants. Values are mean ± SE (n = 7). One or two asterisks indicate significance corresponding to *P < 0.05 or **P < 0.01 (Student t-test). G Ⅰ, Ⅱ, Ⅲ indicate group Ⅰ, Ⅱ, Ⅲ respectively.
(DOC)

S2 Table Forage quality analysis of *STF* transgenic switchgrass. ^a^IVTDMD, in vitro true dry matter digestibility; ^b^ADL, acid detergent lignin. The *STF* transgenic switchgrass and control plants were harvested after 4-month growth in the greenhouse. Values are mean ± SE (n = 3). One or two asterisks indicate significance corresponding to *P < 0.05 or **P < 0.01 (Student t-test).
(DOC)

S3 Table Down-regulated genes in *STF* overexpressing switchgrass lines. Fold change presented as relative abundance of transcript in *STF* overexpression/control (*UBI*::*STF/UBI*::*GUS*) switchgrass plants. P-value calculated as described in materials and methods. “//”, no significant similarity found.
(DOC)

S4 Table Up-regulated genes in *STF* overexpressing switchgrass. Fold change presented as relative abundance of transcript in *STF* overexpression/control (*UBI*::*STF/UBI*::*GUS*) switchgrass plants. P-value calculated as described in materials and methods. “//”, no significant similarity found.
(DOC)

S5 Table Quantification of Cytokinins in *STF* overexpression rice lines. The top two leaf blades of three *UBI*::*STF* transgenic rice lines and wild type at vegetative stage (2 months after planting) were collected for Quantification of Cytokinins. iP, isopentenyladenine; iPR, iP riboside; iP9G, iP 9-glucoside; tZ, zeatin; tZR, zeatin riboside; tZ9G, zeatin 9-glucoside. The unit is ng·g^-1^ FW.
(DOC)

S6 Table Accession numbers of CKX family members in rice, *Brachypodium* and switchgrass. (DOC)

S7 Table Primers used in this study. (DOC)
