# Supplementary figures and images for: Overexpression of the WOX gene STENOFOLIA improves biomass yield and sugar release in transgenic grasses and display altered cytokinin homeostasis
Source: PLoS Genet. 2017 Mar 6;13(3):e1006649. doi: 10.1371/journal.pgen.1006649 (PMC5358894; doi:10.1371/journal.pgen.1006649)

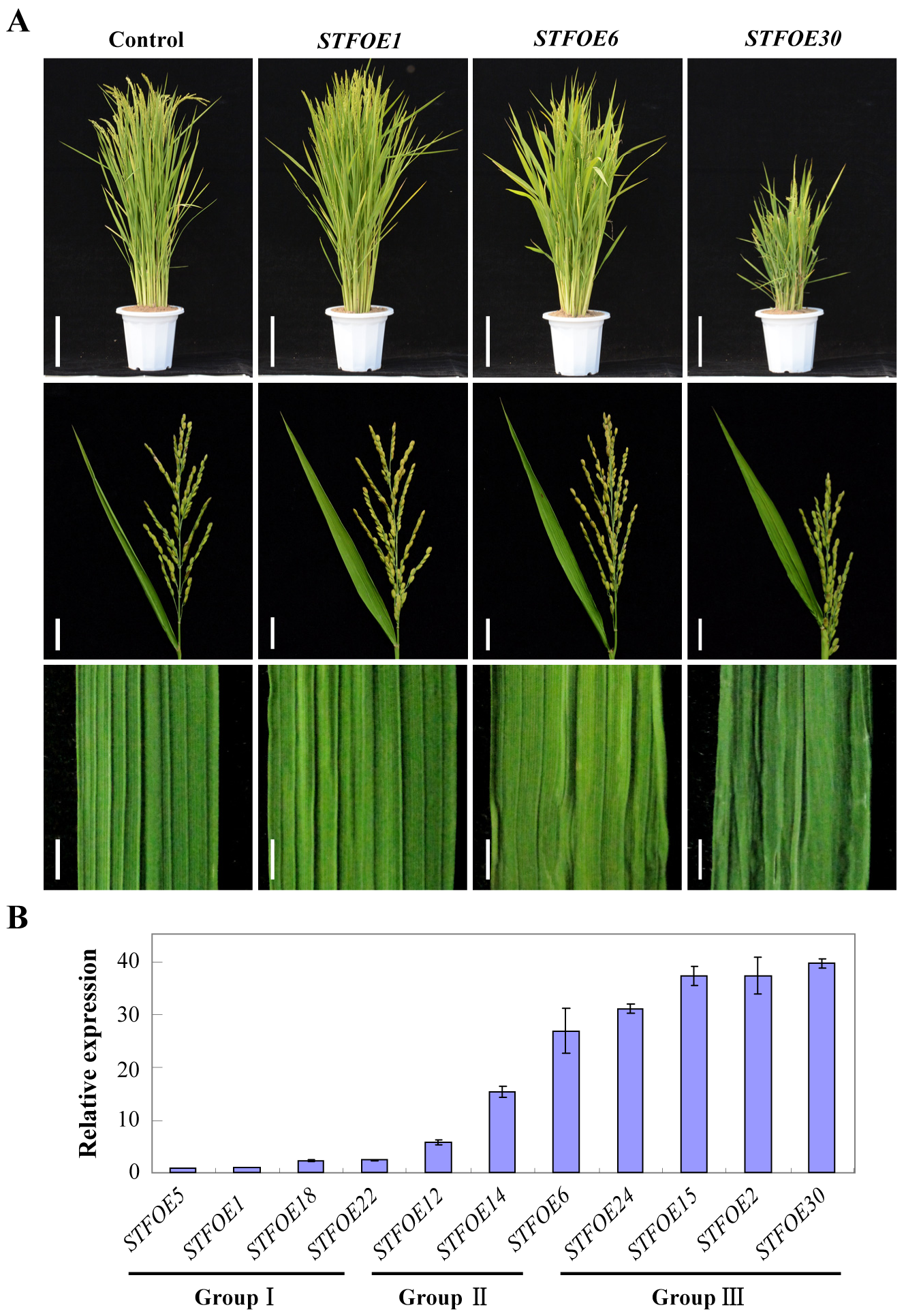

Supplement: S1 Fig — (A) Phenotypes of three classes of STF overexpressing rice lines. The control was transformed in the same way with UBI::GFP. Bars = 10 cm in upper row, 2 cm in middle row and 2 mm in lower row. (B) Transcript abundance of STF in transgenic plants revealed by qRT-PCR. Bars represent means ± SE of three technical replicates. (TIF) [file pgen.1006649.s001.tif]

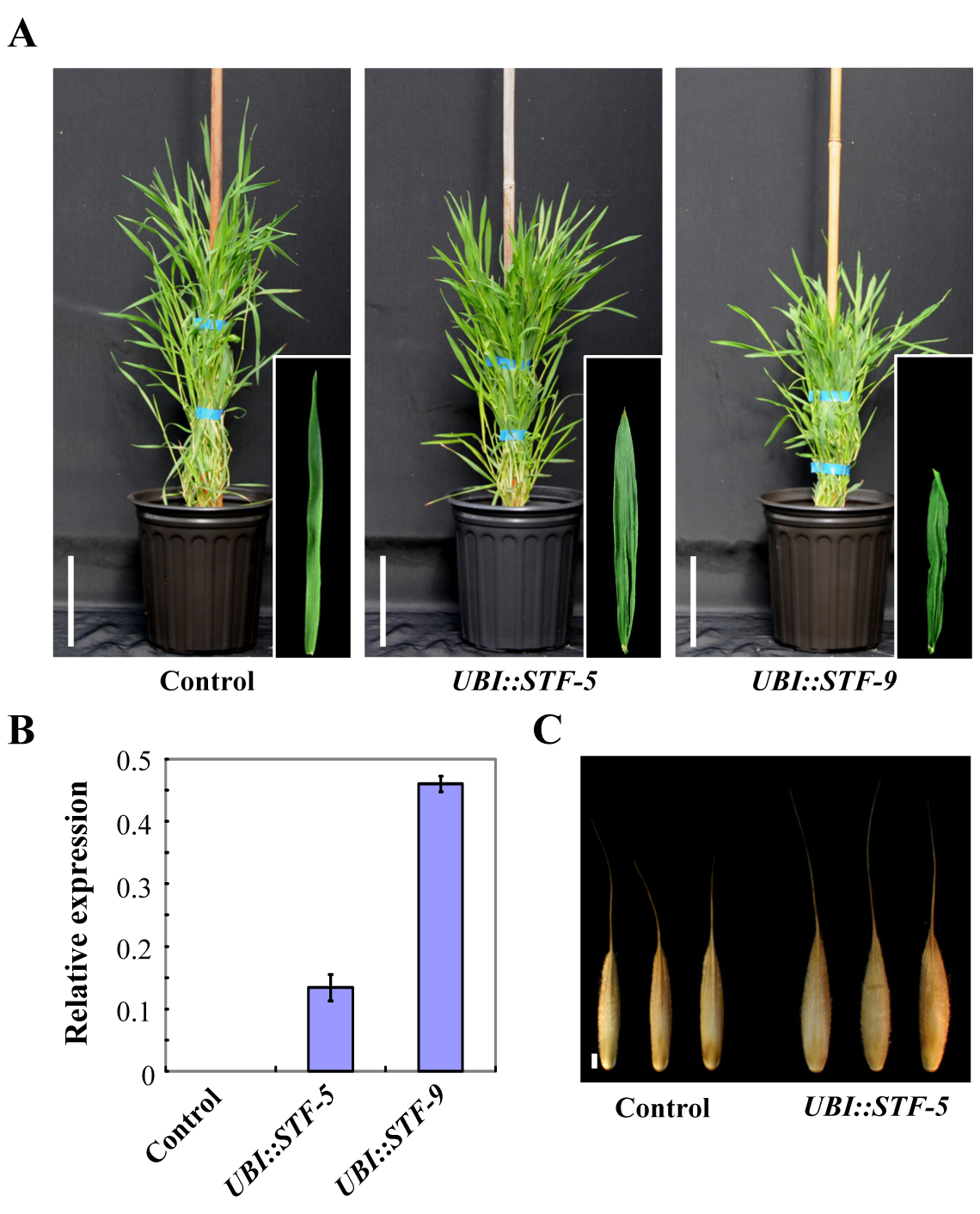

Supplement: S2 Fig — (A) Phenotypes of STF overexpressing Brachypodium. UBI::GUS transformed Brachypodium was used as the control. Bars = 10 cm. (B) Transcript abundance of STF in transgenic plants revealed by qRT-PCR. Bars represent means ± SE of three technical replicates. (C) Comparison of seed size between STF transgenic and control (UBI::GUS) plants. Bar = 1 mm. (TIF) [file pgen.1006649.s002.tif]

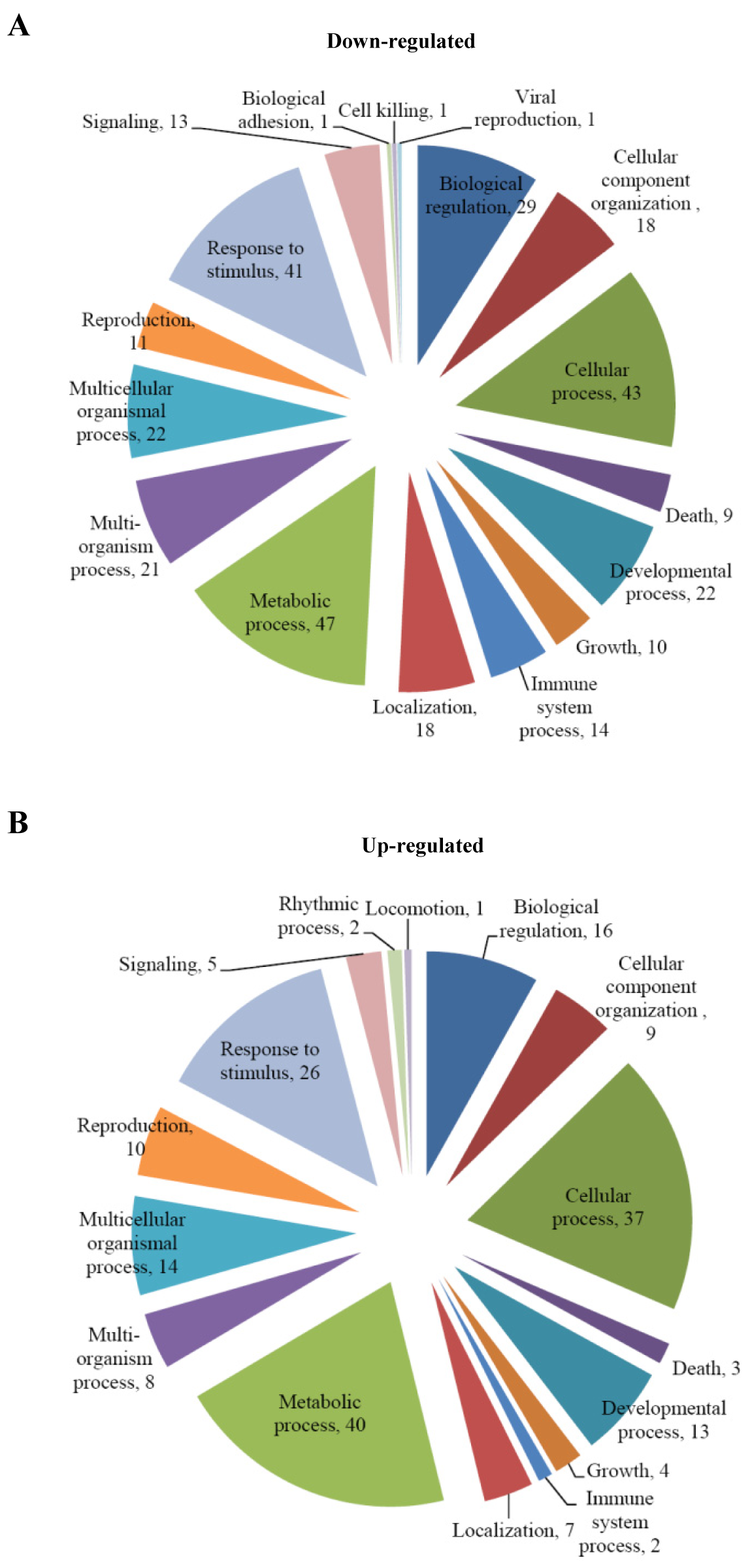

Supplement: S3 Fig — A pie chart representing the distribution of functional classifications of down-regulated (A) and up-regulated (B) probes based on the Gene Ontology Assignments. (TIF) [file pgen.1006649.s003.tif]

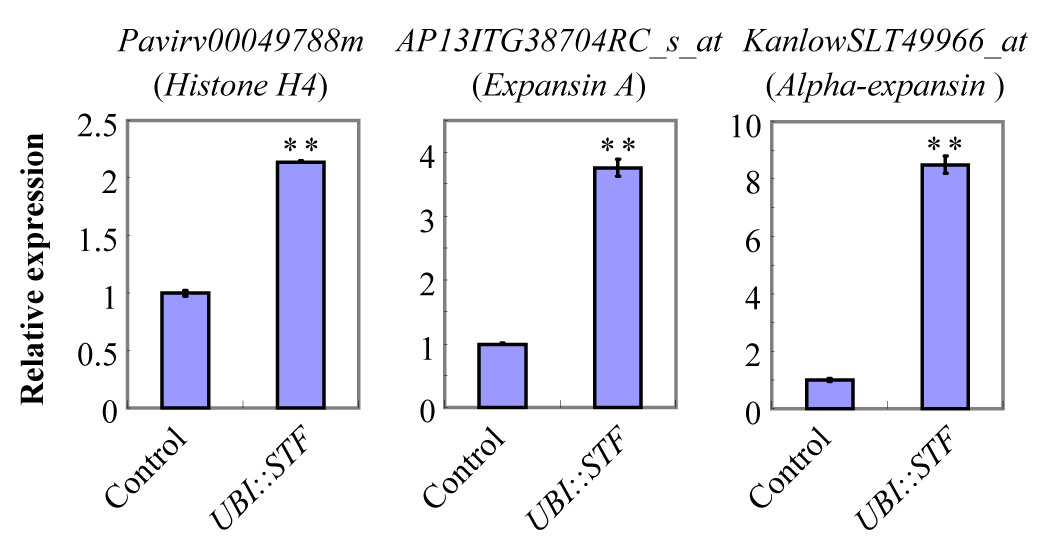

Supplement: S4 Fig — Transcript levels of genes encoding putative Histone H4, Expansin A and Alpha-expansin in STF transgenic lines revealed by qRT-PCR. UBI::GUS expressing switchgrass plants were used as controls. Bars represent means ± SE of three technical replicates and two biological replicates. The asterisks indicate significant differences (** means p < 0.01, Student t-test). (TIF) [file pgen.1006649.s004.tif]

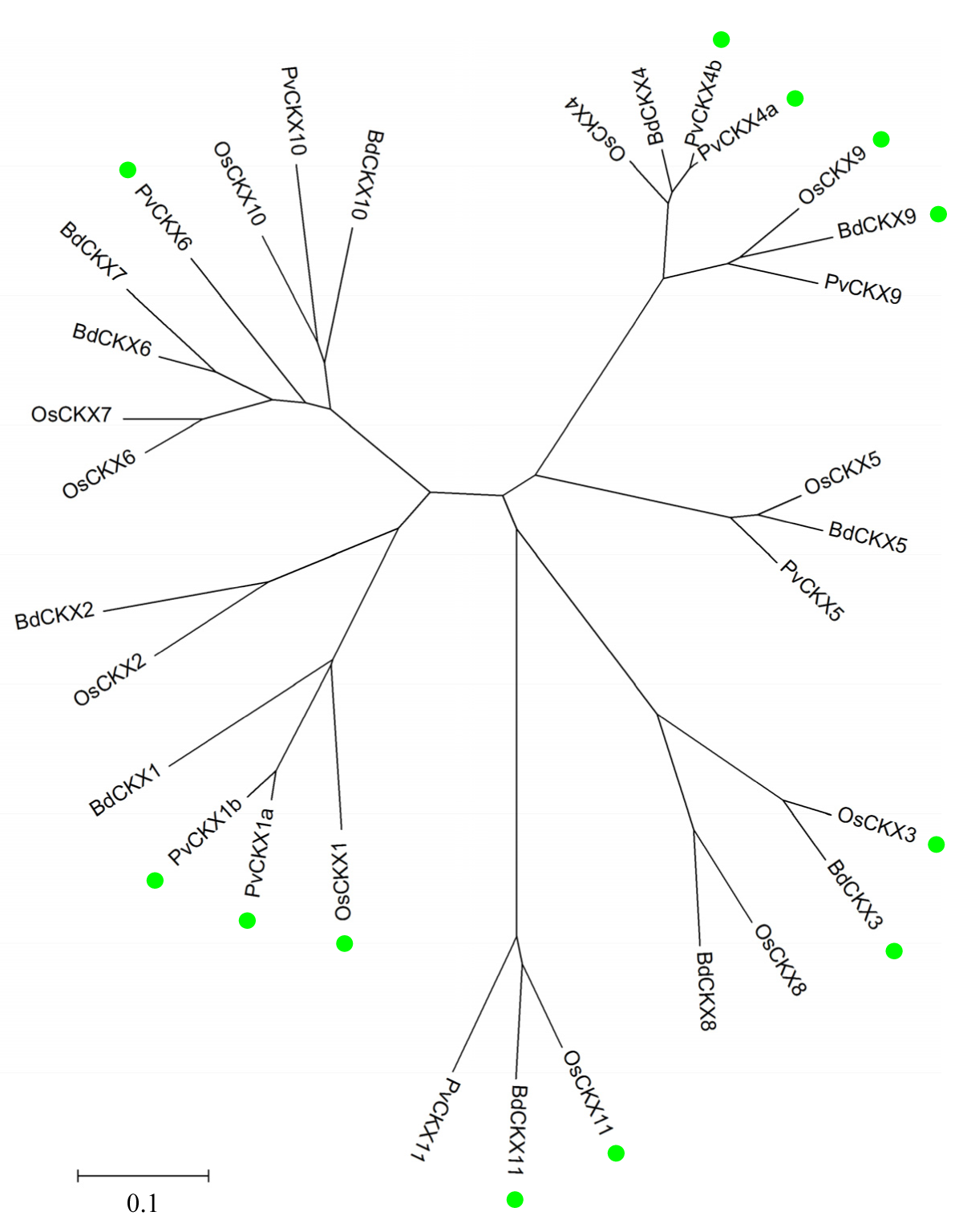

Supplement: S5 Fig — Full-length amino acid sequences were aligned using Clustal W and the tree was constructed using MEGA4 with 1000 replicates. Species: Os, Oryza sativa; Bd, Brachypodium distachyon; Pv, Panicum virgatum. The green dots highlight the downregulated CKXs in STF overexpressing grasses. (TIF) [file pgen.1006649.s005.tif]

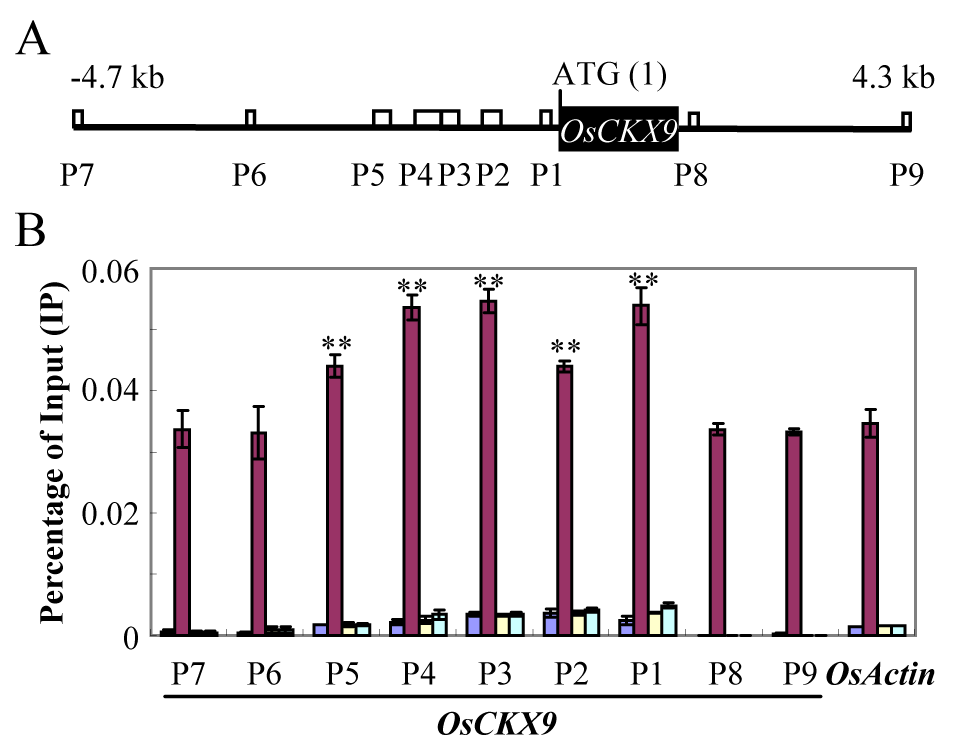

Supplement: S6 Fig — (A) Schematic representation of the regions of OsCKX9 tested by ChIP experiments. P1-P5 are specific STF binding sites, while P6-P9 are non-specific sites used as control. (B) ChIP assay showing the association of STF with several regions in the promoter of OsCKX9 (P1-P5) compared to background signal (P6-P9) or the OsActin negative control. Bars represent means ± SE of three technical replicates and two biological replicates. The asterisks indicate significant differences (** p < 0.01, Student t-test). (TIF) [file pgen.1006649.s006.tif]

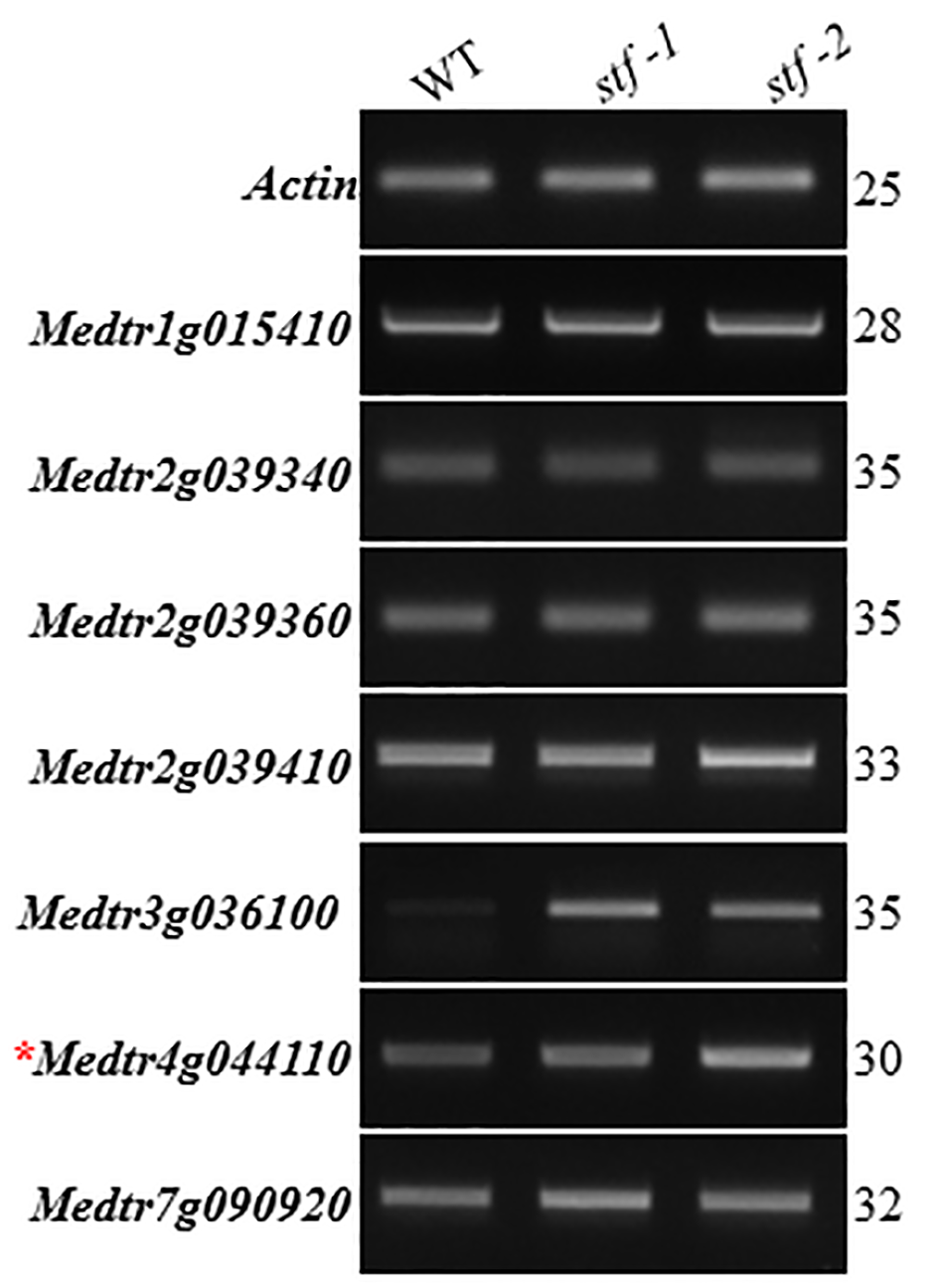

Supplement: S7 Fig — Semi quantitative RT-PCR showing the expression of seven CKX genes in the leaves of four weeks old M. truncatula stf mutants compared to wild type. Numbers on the right show the number of PCR cycles used. * represents a gene that showed weak induction in stf microarray. (TIF) [file pgen.1006649.s007.tif]
