# Supplementary material for: Overexpression of the WOX gene STENOFOLIA improves biomass yield and sugar release in transgenic grasses and display altered cytokinin homeostasis
Source: PLoS Genet. 2017 Mar 6;13(3):e1006649. doi: 10.1371/journal.pgen.1006649 (PMC5358894; doi:10.1371/journal.pgen.1006649)
Supplement: S1 Table — Plant height and tiller number of STF transgenic and control switchgrass plants were measured after 4-months of growth in the greenhouse. 7 tillers were used to measure internode length and diameter (internode Ⅱ), leaf blade length and width were measured in the 3rd leaf for each plant. The control represents the average of three independent UBI::GUS transgenic plants. Values are mean ± SE (n = 7). One or two asterisks indicate significance corresponding to *P < 0.05 or **P < 0.01 (Student t-test). G Ⅰ, Ⅱ, Ⅲ indicate group Ⅰ, Ⅱ, Ⅲ respectively. (DOC) [file pgen.1006649.s008.doc]

| **Transgenic**  **lines** | **Plant**  **height**  **(cm)** | **Range of internode number** | | **Internode length**  **(cm)** | **Internode diameter (mm)** | **Leaf length**  **(cm)** | **Leaf width**  **(mm)** | **Total**  **tiller number** | | **Flowering time**  **(day)** | |
| --- | --- | --- | --- | --- | --- | --- | --- | --- | --- | --- | --- |
| **Control** | 129.7± 1.6 | | 5-7 | 8.6 ± 0.4 | 3.2 ± 0.1 | 30.8 ± 0.4 | 10.1 ± 0.2 | | 46±3 | | 92 ± 1.2 |
| **STF-16（G** Ⅰ**）** | 128.9 ± 3.4 | | 5-6 | 12.7 ± 1.4* | 4 ± 0.2* | 31.1 ± 0.5 | 13.9 ± 0.2** | | 50 | | 92 ± 2 |
| **STF-17（G** Ⅰ**）** | 127.9 ± 1.5 | | 5-7 | 9.5 ± 1 | 4. ± 0.2* | 30.2 ± 0.6 | 12.7 ± 0.3** | | 54 | | 91 ± 1.8 |
| **STF-21（G** Ⅰ**）** | 129.4 ± 1.6 | | 5-7 | 9.3 ± 0.7 | 3.8 ± 0.1* | 30.1 ± 0.6 | 12.1 ± 0.3* | | 54 | | 90 ± 1.8 |
| **STF-2 （G** Ⅱ**）** | 137.1± 3.4 | | 5-7 | 11.7 ± 0.8 | 4.8 ± 0.3** | 31.1 ± 0.6 | 15.8 ± 0.5** | | 57 | | 89 ± 1.9 |
| **STF-4 （G** Ⅱ**）** | 139.3 ± 3.2 | | 5-6 | 12.9 ± 0.8* | 5.1 ± 0.3** | 30.9 ± 0.7 | 15.4 ± 0.4** | | 53 | | 89 ± 1.8 |
| **STF-10（G** Ⅱ**）** | 145.2 ± 3.1* | | 5-6 | 13.7 ± 1.4** | 4.6 ± 0.1** | 32.2 ± 1.1 | 14.1 ± 0.3** | | 50 | | 89 ± 1.8 |
| **STF-9 （G** Ⅲ**）** | 75.9 ± 5.1** | | 3-5 | 11 ± 1.2 | 4.4 ± 0.2** | 24.2 ± 0.9** | 14.4 ± 0.6** | | 47 | | >130 |
| **STF-12（G** Ⅲ**）** | 60.4 ± 3.9** | | 2-5 | 8.4 ± 0.6 | 4.2 ± 0.2* | 23.1 ± 1.6** | 15.2 ± 0.7** | | 40 | | >130 |
