# Supplementary material for: Overexpression of the WOX gene STENOFOLIA improves biomass yield and sugar release in transgenic grasses and display altered cytokinin homeostasis
Source: PLoS Genet. 2017 Mar 6;13(3):e1006649. doi: 10.1371/journal.pgen.1006649 (PMC5358894; doi:10.1371/journal.pgen.1006649)
Supplement: S2 Table — aIVTDMD, in vitro true dry matter digestibility; bADL, acid detergent lignin. The STF transgenic switchgrass and control plants were harvested after 4-month growth in the greenhouse. Values are mean ± SE (n = 3). One or two asterisks indicate significance corresponding to *P < 0.05 or **P < 0.01 (Student t-test). (DOC) [file pgen.1006649.s009.doc]

| **Transgenic lines** | **IVTDMD^a^**  **(%)** | **Cellulose**  **(mg/g DW)** | **Hemicellulose**  **(mg/g DW)** | **ADL^b^**  **(mg/g DW)** | **Crude protein**  **(mg/g DW)** |
| --- | --- | --- | --- | --- | --- |
| **Control** | 66.6±0.3 | 269.0±8.3 | 296.8±1.4 | 72.0±1.3 | 126.5±6.3 |
| **Group Ⅰ** | 68.0±0.2 | 252.2±3.7 | 294.3±3.1 | 73.6±0.5 | 143.4±3.8 |
| **Group Ⅱ** | 69.3±0.8* | 247.1±3.9 | 287.7±2.4 | 73.2±1.0 | 156.8±4.0* |
| **Group Ⅲ** | 71.4±0.3** | 232.5±2.1* | 275.9±0.1** | 66.8±1.1** | 174.3±1.3** |
