# Supplementary material for: Overexpression of the WOX gene STENOFOLIA improves biomass yield and sugar release in transgenic grasses and display altered cytokinin homeostasis
Source: PLoS Genet. 2017 Mar 6;13(3):e1006649. doi: 10.1371/journal.pgen.1006649 (PMC5358894; doi:10.1371/journal.pgen.1006649)
Supplement: S3 Table — Fold change presented as relative abundance of transcript in STF overexpression/control (UBI::STF/UBI::GUS) switchgrass plants. P-value calculated as described in materials and methods. “//”, no significant similarity found. (DOC) [file pgen.1006649.s010.doc]

| **Probeset ID** | **CDD annotation** | **Fold change** | **P-value** |
| --- | --- | --- | --- |
| AP13ITG53058_at | cd01803/Ubiquitin | 0.005 | 0.01273 |
| AP13ITG63551_s_at | // | 0.011 | 0.00261 |
| OTHSWCTG20217_at | // | 0.013 | 0.00003 |
| KanlowCTG32251_at | PLN00116/translation elongation factor EF-2 subunit | 0.022 | 0.02324 |
| KanlowCTG39289_at | PLN02173/UDP-glucosyl transferase family protein | 0.022 | 0.26453 |
| AP13ITG71453_at | // | 0.023 | 0.02174 |
| AP13ITG39062_x_at | cd01803/Ubiquitin | 0.025 | 0.00381 |
| AP13ITG56782_s_at | // | 0.025 | 0.01412 |
| KanlowCTG35790_at | // | 0.026 | 0.00011 |
| AP13ITG70897_at | PLN02164/sulfotransferase family protein | 0.027 | 0.04890 |
| AP13ITG50363_at | PLN02381/aminoacyl-tRNA ligase | 0.035 | 0.01495 |
| KanlowCTG17037_s_at | pfam08523/Multiprotein bridging factor 1 | 0.038 | 0.01143 |
| AP13CTG24979_s_at |  | 0.038 | 0.02127 |
| AlamoCTG05339_s_at | // | 0.038 | 0.00001 |
| AP13CTG56865RC_at | cd05833/Ribosomal protein P2 | 0.039 | 0.01502 |
| AP13ITG57724_at | cd01803/Ubiquitin | 0.039 | 0.02783 |
| OTHSWCTG06795_at | // | 0.041 | 0.36754 |
| KanlowCTG35790_s_at | // | 0.042 | 0.00002 |
| AP13ITG74171_at | PRK12678/transcription termination factor Rho | 0.044 | 0.02120 |
| AP13ITG38511_s_at | PLN02555/limonoid glucosyltransferase | 0.052 | 0.08126 |
| AlamoCTG05276_at | pfam03101/FAR1 DNA-binding domain | 0.052 | 0.00312 |
| KanlowCTG47259RC_at | PLN02534/UDP-glycosyltransferase | 0.053 | 0.31398 |
| KanlowCTG23335_at | pfam10252/Casein kinase substrate phosphoprotein PP28 | 0.054 | 0.01519 |
| OTHSWCTG21028_s_at | // | 0.059 | 0.00573 |
| AP13ITG65101_at | pfam02956/TT viral orf 1. TT virus (TTV) | 0.059 | 0.05809 |
| KanlowCTG21021_at | // | 0.061 | 0.15431 |
| AP13CTG30912_at | PLN02534/UDP-glycosyltransferase | 0.061 | 0.05525 |
| OTHSWCTG15185_at | PLN02511/hydrolase | 0.066 | 0.00166 |
| AP13CTG07265RC_at | pfam00004/ATPase family | 0.073 | 0.07555 |
| KanlowCTG13849_at | PLN02555/limonoid glucosyltransferase | 0.073 | 0.10688 |
| AP13CTG02927_at | pfam01073/3-beta hydroxysteroid dehydrogenase | 0.075 | 0.00228 |
| VS16ITG24577_s_at | pfam02956/TT viral orf 1. TT virus (TTV) | 0.077 | 0.11810 |
| OTHSWCTG12020_s_at | pfam00004/ATPase family | 0.077 | 0.10548 |
| AP13ITG53959_at | PRK12678/transcription termination factor Rho | 0.077 | 0.00529 |
| AlamoCTG08069_at | // | 0.079 | 0.00020 |
| OTHSWSLT37079_at | COG0534/Na+-driven multidrug efflux pump | 0.082 | 0.01081 |
| AP13ITG51564_s_at | pfam01073/3-beta hydroxysteroid dehydrogenase family | 0.088 | 0.00072 |
| AP13CTG18802_at | PLN02286/arginine-tRNA ligase | 0.090 | 0.03166 |
| AP13ITG37706_at | cd00204/ankyrin repeats | 0.094 | 0.00081 |
| AP13ITG63925_s_at | cd01803/Ubiquitin | 0.097 | 0.02418 |
| AP13CTG48112_s_at | // | 0.100 | 0.00005 |
| KanlowCTG46215_s_at | cd04481/RPA1_DBD_B_like | 0.100 | 0.00342 |
| KanlowCTG46713_s_at | // | 0.100 | 0.00047 |
| AP13ITG61578_at | COG1670/Acetyltransferase | 0.102 | 0.13339 |
| KanlowCTG15786_at | cd03185/GST_C family, Class Tau subfamily | 0.104 | 0.19333 |
| KanlowCTG11662_at | PRK12678/transcription termination factor Rho | 0.104 | 0.00161 |
| AP13ITG50362_at | PLN02381/aminoacyl-tRNA ligase | 0.104 | 0.00974 |
| KanlowCTG09824_s_at | COG0534/Na+-driven multidrug efflux pump | 0.106 | 0.00664 |
| AP13CTG07367_at | COG0667/Predicted oxidoreductases | 0.107 | 0.12918 |
| KanlowCTG11199_at | PLN00043/elongation factor 1-alpha | 0.109 | 0.00111 |
| AP13ITG63455_at | pfam02956/TT viral orf 1. TT virus (TTV) | 0.112 | 0.02509 |
| AP13ITG59377_s_at | PLN02290/cytokinin trans-hydroxylase | 0.112 | 0.01188 |
| AP13ITG40604_s_at | // | 0.114 | 0.12352 |
| AlamoCTG14387_s_at | // | 0.116 | 0.01958 |
| AP13ITG69978_at | pfam12004/Domain of unknown function (DUF3498) | 0.119 | 0.05228 |
| AP13ITG67384-RC_at |  | 0.119 | 0.12155 |
| AP13ITG57421RC_at | pfam02458/Transferase family | 0.120 | 0.01728 |
| AP13ITG63913_at | // | 0.123 | 0.17797 |
| OTHSWCTG12305_s_at | // | 0.126 | 0.08683 |
| OTHSWCTG06395_s_at | // | 0.126 | 0.00091 |
| AP13CTG14697_s_at | pfam00069/Protein kinase domain. | 0.128 | 0.11887 |
| OTHSWCTG21992_s_at | pfam03004/Plant transposase (Ptta/En/Spm family) | 0.133 | 0.00201 |
| AP13ITG52422_s_at | PTZ00141/elongation factor 1 alpha | 0.133 | 0.00483 |
| AP13CTG20898_x_at | PLN02183/ferulate 5-hydroxylase | 0.135 | 0.14259 |
| AP13CTG25213_at | // | 0.135 | 0.02077 |
| KanlowCTG33522_s_at | // | 0.135 | 0.10061 |
| AP13ITG37705_s_at | cd00204/ankyrin repeats | 0.136 | 0.00597 |
| AP13CTG48044_s_at | // | 0.136 | 0.00152 |
| AP13CTG25288_at | cd06144/RNA exonuclease | 0.137 | 0.03556 |
| KanlowCTG40733_s_at | // | 0.139 | 0.00166 |
| AP13CTG05226_at | pfam07727/Reverse transcriptase | 0.139 | 0.00037 |
| KanlowCTG08343_s_at | cd00684/Plant Terpene Cyclases | 0.140 | 0.04527 |
| AP13CTG09529_at | PLN02173/UDP-glucosyl transferase family protein | 0.141 | 0.09407 |
| AP13ITG66548_s_at | PRK05557/3-ketoacyl-(acyl-carrier-protein) reductase | 0.144 | 0.16819 |
| KanlowCTG00735_s_at | PLN00043/elongation factor 1-alpha; Provisional | 0.148 | 0.00100 |
| AP13ITG73908_at | // | 0.148 | 0.35364 |
| AP13ITG76352-RC_at | // | 0.150 | 0.00677 |
| AP13CTG25829_at | // | 0.152 | 0.02197 |
| AP13CTG08928_s_at | pfam07690/Major Facilitator Superfamily | 0.152 | 0.02636 |
| KanlowCTG38713_s_at | PLN02534/UDP-glycosyltransferase | 0.153 | 0.13103 |
| AP13ITG52422_at | PTZ00141/elongation factor 1 alpha | 0.156 | 0.00821 |
| AlamoCTG09684_at | APETALA2 like DNA-binding domain protein | 0.156 | 0.01392 |
| KanlowSLT52764_at | pfam07727/Reverse transcriptase | 0.157 | 0.00108 |
| AP13ITG53181RC_at | PRK12270/alpha-ketoglutarate decarboxylase | 0.158 | 0.03128 |
| AP13CTG03477_s_at | cd00684/Plant Terpene Cyclases, Class 1 | 0.159 | 0.03324 |
| AP13CTG05506_s_at | PLN02534/UDP-glycosyltransferase | 0.162 | 0.12581 |
| AP13CTG15902_s_at | cd00684/Plant Terpene Cyclases, Class 1 | 0.162 | 0.04477 |
| AP13ITG49094_s_at | pfam00004/ATPase family | 0.165 | 0.02682 |
| OTHSWCTG06541_s_at | // | 0.165 | 0.00017 |
| AP13ITG71440_at | PLN02183/ferulate 5-hydroxylase | 0.168 | 0.16208 |
| KanlowCTG45440_s_at | pfam03222/Tryptophan/tyrosine permease family | 0.168 | 0.08484 |
| AP13CTG05942_s_at | PLN02321/2-isopropylmalate synthase | 0.171 | 0.01513 |
| AP13CTG05811RC_at | pfam00004/ATPase family | 0.171 | 0.03873 |
| KanlowCTG09763_at | Chloroplast Nucleoids DNA-binding Protease | 0.171 | 0.01594 |
| AP13ITG77248_s_at | // | 0.174 | 0.25639 |
| OTHSWCTG09574_s_at | // | 0.176 | 0.22204 |
| KanlowCTG10507_at | cd01040/Globin | 0.179 | 0.37603 |
| AP13CTG17622_at | Polygalacturonate 4-alpha-galacturonosyltransferase | 0.179 | 0.01014 |
| AP13CTG55038_at | // | 0.180 | 0.14155 |
| AP13ITG70986_at | TIGR01160/translation initiation factor SUI1 | 0.180 | 0.01437 |
| AP13ITG42090_s_at | // | 0.180 | 0.07626 |
| AP13CTG24692_at | pfam00004/ATPase family | 0.184 | 0.11879 |
| AP13ITG58535_at | pfam00332/Glycosyl hydrolases family | 0.185 | 0.17700 |
| VS16ITG10369_at | // | 0.185 | 0.00215 |
| KanlowSLT51889_s_at | PHA03245/large tegument protein UL36 | 0.185 | 0.02273 |
| AP13ITG59377_at | PLN02290/cytokinin trans-hydroxylase | 0.186 | 0.01268 |
| AlamoCTG09225_at | // | 0.186 | 0.03287 |
| OTHSWSLT37030_at | // | 0.188 | 0.01844 |
| AP13ITG60452_at | Xylanase inhibitor Xip-I, | 0.188 | 0.13768 |
| AP13CTG22460_at | Non-specific lipid-transfer protein-like subfamily | 0.189 | 0.34815 |
| VS16ITG12570_at | // | 0.192 | 0.00776 |
| OTHSWCTG18839_at | // | 0.194 | 0.00242 |
| OTHSWSLT27671_at | // | 0.195 | 0.00002 |
| OTHSWCTG20479_at | PRK05557/3-ketoacyl-(acyl-carrier-protein) reductase | 0.195 | 0.21289 |
| AlamoCTG13165_at | // | 0.195 | 0.00949 |
| OTHSWSLT27780_at | PLN02670/transferase, transferring glycosyl groups | 0.196 | 0.28813 |
| AP13CTG17008_at | cd01803/Ubiquitin | 0.196 | 0.00835 |
| AlamoCTG14108_at | pfam04937/Protein of unknown function (DUF 659) | 0.197 | 0.00000 |
| AP13ITG62754_s_at | COG0412/Dienelactone hydrolase and related enzymes | 0.201 | 0.06696 |
| KanlowCTG20096_at | // | 0.201 | 0.19441 |
| KanlowCTG28441_at | // | 0.203 | 0.00098 |
| AP13ITG76718_at | PHA03247/large tegument protein UL36 | 0.204 | 0.00181 |
| KanlowCTG26345_at | // | 0.204 | 0.26168 |
| AP13CTG07495_s_at | pfam06658/Protein of unknown function (DUF1168) | 0.205 | 0.00794 |
| AP13ITG59757_s_at | PRK08177/short chain dehydrogenase | 0.207 | 0.20546 |
| KanlowCTG20829_s_at | pfam00574/Clp protease | 0.208 | 0.02651 |
| AP13ITG42090_at | // | 0.208 | 0.05138 |
| KanlowCTG42502_at | pfam04484/Family of unknown function (DUF566) | 0.210 | 0.02670 |
| AP13CTG04816_s_at | pfam00955/HCO3- transporter family | 0.210 | 0.00238 |
| AP13ITG76266_at | S-adenosylmethionine-dependent methyltransferase | 0.211 | 0.30658 |
| AP13ITG74837_at | PLN00164/glucosyltransferase | 0.212 | 0.18439 |
| AP13ITG66311_at | // | 0.213 | 0.06742 |
| AlamoCTG02811_at | pfam00665/Integrase core domain | 0.213 | 0.01065 |
| AP13ITG73237_at | // | 0.214 | 0.13744 |
| AP13ITG71976_at | PRK12270/alpha-ketoglutarate decarboxylase | 0.216 | 0.03072 |
| AP13CTG30063_at | pfam00069/Protein kinase domain | 0.219 | 0.11432 |
| AP13ITG56605_at | pfam08615/Ribonuclease H2 non-catalytic subunit | 0.219 | 0.00103 |
| AP13CTG50688_s_at | smart00220/Serine/Threonine protein kinase | 0.219 | 0.13331 |
| KanlowCTG09897_x_at | cd01926/cyclophilin_ABH_like | 0.221 | 0.01095 |
| AP13ITG66346-RC_at | // | 0.221 | 0.00500 |
| AP13CTG24953RC_at | PLN02534/UDP-glycosyltransferase | 0.222 | 0.00722 |
| AP13CTG25175_at | PHA03247/large tegument protein UL36 | 0.222 | 0.17757 |
| KanlowCTG47289_s_at | PRK07538/hypothetical protein | 0.223 | 0.17686 |
| KanlowCTG46140_at | // | 0.223 | 0.14984 |
| AP13ITG59602_at | // | 0.225 | 0.00226 |
| KanlowCTG12932_s_at | PLN02183/ferulate 5-hydroxylase | 0.226 | 0.13876 |
| AP13ITG58367_at | // | 0.226 | 0.13557 |
| KanlowCTG39635_at | pfam00067/Cytochrome P450 | 0.226 | 0.08454 |
| AP13CTG26173_at | Cytochrome b5-like Heme/Steroid binding domain | 0.227 | 0.18867 |
| AP13ITG52395_s_at | cd03185/GST_C family, Class Tau subfamily | 0.227 | 0.00097 |
| KanlowCTG09678_s_at | Pleiotropic Drug Resistance (PDR) Family protein | 0.230 | 0.00431 |
| KanlowCTG23007_at | // | 0.231 | 0.18754 |
| AlamoCTG11165_s_at | // | 0.232 | 0.03920 |
| AP13CTG30063_s_at | pfam00069/Protein kinase domain | 0.232 | 0.08652 |
| AP13ITG63673-RC_at | // | 0.232 | 0.13847 |
| AP13ITG75162_s_at | // | 0.233 | 0.00129 |
| AP13ITG69458_at | pfam01190/Pollen proteins Ole e I like | 0.234 | 0.07239 |
| AP13ITG77184_at | PLN02173/UDP-glucosyl transferase family protein | 0.234 | 0.35973 |
| AlamoCTG02199_at | PRK09138/DNA topoisomerase I | 0.234 | 0.02483 |
| KanlowCTG05050_s_at | Transmembrane amino acid transporter protein | 0.235 | 0.07128 |
| KanlowCTG27752_at | COG0667/Predicted oxidoreductase | 0.235 | 0.19102 |
| AP13CTG58862_s_at | pfam00504/Chlorophyll A-B binding protein | 0.236 | 0.07918 |
| AP13CTG34009_s_at | PLN02849/glycosyl hydrolase family 1 protein | 0.236 | 0.09919 |
| AP13ITG60526_at | pfam05110/AF-4 proto-oncoprotein | 0.236 | 0.08938 |
| KanlowSLT53081_x_at | // | 0.237 | 0.00031 |
| AP13CTG31837_s_at | // | 0.237 | 0.00940 |
| KanlowCTG08043_s_at | pfam00504/Chlorophyll A-B binding protein. | 0.238 | 0.16979 |
| AP13CTG31279RC_at | pfam00504/Chlorophyll A-B binding protein. | 0.238 | 0.05353 |
| AlamoCTG08796_at | // | 0.238 | 0.00828 |
| KanlowCTG29333_at | // | 0.238 | 0.12483 |
| AP13ITG72285_s_at | // | 0.238 | 0.14347 |
| OTHSWSLT36401_s_at | Multi drug resistance-associated protein (MRP) | 0.240 | 0.00922 |
| KanlowCTG26335_at | pfam00314/Thaumatin family | 0.240 | 0.15335 |
| AP13CTG17815_at | PRK12438/hypothetical protein | 0.240 | 0.00226 |
| AP13CTG07180_s_at | PLN02361/alpha-amylase | 0.241 | 0.01681 |
| AP13ITG73696_at | cd02877/xylanase inhibitor Xip-I | 0.241 | 0.13724 |
| VS16ITG21051_at | // | 0.241 | 0.01691 |
| AP13CTG18609_at | cd03203/GST_C family, Class Lambda subfamily | 0.241 | 0.25042 |
| AP13ITG55927-RC_at | // | 0.241 | 0.10706 |
| KanlowCTG10053_s_at | cd00684/Plant Terpene Cyclase | 0.242 | 0.05572 |
| KanlowCTG23219_s_at | // | 0.242 | 0.00909 |
| KanlowCTG29975_s_at | pfam00504/Chlorophyll A-B binding protein | 0.244 | 0.07707 |
| AP13ITG60479_s_at | PHA03245/large tegument protein UL36 | 0.246 | 0.11527 |
| OTHSWCTG20654_at | // | 0.246 | 0.00033 |
| OTHSWCTG21216_s_at | // | 0.248 | 0.02376 |
| AP13CTG50688_at | smart00220/Serine/Threonine protein kinase | 0.248 | 0.14561 |
| AP13CTG16606_s_at | cd03203/GST_C family | 0.249 | 0.06824 |
| AP13CTG15102_s_at | Pleiotropic Drug Resistance (PDR) Family protein | 0.250 | 0.10517 |
| OTHSWCTG19883_at | pfam00069/Protein kinase domain | 0.251 | 0.09680 |
| KanlowCTG33522_at | // | 0.251 | 0.28399 |
| AP13ITG73916_at | // | 0.251 | 0.13804 |
| AP13ITG42000_at | pfam00314/Thaumatin family | 0.251 | 0.15155 |
| AP13ITG67654_at | PLN02992/coniferyl-alcohol glucosyltransferase | 0.252 | 0.03991 |
| KanlowCTG02782_s_at | // | 0.253 | 0.07424 |
| AP13CTG13652_s_at | TIGR00792/sugar transporter | 0.256 | 0.15010 |
| KanlowCTG06582RC_at | PLN02555/limonoid glucosyltransferase | 0.256 | 0.03334 |
| KanlowCTG40962_at | PLN02500/steroid 22alpha-hydroxylase | 0.256 | 0.00200 |
| AP13ITG52053_s_at | Hydrolase, hydrolyzing O-glycosyl compounds | 0.258 | 0.23922 |
| AP13ITG62990_at | pfam00314/Thaumatin family | 0.260 | 0.21409 |
| AP13CTG16611_at | PRK09354/recombinase A | 0.260 | 0.02387 |
| AP13ITG67309_s_at | pfam00450/Serine carboxypeptidase | 0.260 | 0.22462 |
| AP13ITG63170_at | pfam03018/Dirigent-like protein | 0.260 | 0.00153 |
| KanlowCTG34953_s_at | // | 0.261 | 0.25834 |
| KanlowCTG08097_s_at | PHA03245/large tegument protein UL36 | 0.261 | 0.01632 |
| AP13CTG53852_at | TIGR03083/uncharacterized Actinobacterial protein | 0.262 | 0.16279 |
| AP13ITG49578_at | cd02667/A subfamily of Peptidase C19 | 0.262 | 0.00422 |
| AP13ITG75527_s_at | // | 0.264 | 0.32894 |
| AP13ITG65227_at | // | 0.266 | 0.00049 |
| KanlowCTG46145_s_at | cd05579/Serine/threonine kinase-like protein | 0.266 | 0.04929 |
| KanlowCTG09229_at | DNA polymerase III subunits gamma and tau | 0.267 | 0.00158 |
| AP13ITG76703_at | // | 0.268 | 0.00997 |
| AP13ITG37277_s_at | COG0534/Na+-driven multidrug efflux pump | 0.269 | 0.00788 |
| AP13ITG71171RC_s_at | PLN02534/UDP-glycosyltransferase | 0.269 | 0.29981 |
| KanlowCTG08567_s_at | pfam00230/Major intrinsic protein | 0.269 | 0.13105 |
| AP13ITG39117_s_at | // | 0.269 | 0.05329 |
| KanlowCTG34086_at | // | 0.269 | 0.01985 |
| AP13ITG73883_s_at | // | 0.269 | 0.01281 |
| KanlowCTG42804_s_at | DNA polymerase III subunits gamma and tau | 0.270 | 0.15865 |
| KanlowCTG29377_at | // | 0.270 | 0.00510 |
| AP13ITG61014_at | pfam12609/Wound-induced protein | 0.270 | 0.07931 |
| KanlowSLT54444_s_at | // | 0.273 | 0.09231 |
| AP13ITG55437_at | cd03185/GST_C family, Class Tau subfamily | 0.274 | 0.13974 |
| OTHSWCTG13359_at | PRK13057/putative lipid kinase | 0.274 | 0.05039 |
| AP13CTG29259_at | COG0265/Trypsin-like serine protease | 0.274 | 0.01234 |
| AP13CTG07636_at | Transmembrane amino acid transporter protein | 0.274 | 0.06998 |
| KanlowCTG44446_at | Non-LTR retrovirus reverse transcriptase | 0.275 | 0.00022 |
| KanlowCTG26431_s_at | // | 0.276 | 0.02133 |
| KanlowCTG17861_s_at | PRK12678/transcription termination factor Rho | 0.277 | 0.01711 |
| AP13CTG37537_s_at | // | 0.277 | 0.24182 |
| AP13CTG09468_at | smart00219/Tyrosine kinase, catalytic domain | 0.277 | 0.14049 |
| AlamoCTG14328_at | Transmission-blocking target antigen s230 | 0.278 | 0.00671 |
| AP13ITG67741_at | pfam00182/Chitinase class I | 0.278 | 0.15760 |
| VS16ITG01784_s_at | TIGR00887/phosphate:H+ symporter | 0.278 | 0.10343 |
| AP13ITG75785RC_at | PLN02173/UDP-glucosyl transferase family protein | 0.279 | 0.10379 |
| AP13CTG32336_at | // | 0.280 | 0.01755 |
| AP13ITG62654_at | // | 0.282 | 0.00890 |
| KanlowCTG21193_at | PHA03245/large tegument protein UL36 | 0.282 | 0.00789 |
| AP13ITG69514_s_at | PLN02687/flavonoid 3'-monooxygenase | 0.282 | 0.02789 |
| OTHSWCTG01972_at | pfam05078/Protein of unknown function (DUF679) | 0.282 | 0.08816 |
| AP13CTG03484_at | smart00219/Tyrosine kinase, catalytic domain | 0.283 | 0.13818 |
| OTHSWCTG15870_at | pfam03106/WRKY DNA -binding domain | 0.283 | 0.12671 |
| AP13CTG32494_s_at | // | 0.283 | 0.00235 |
| KanlowCTG20149_at | PLN02972/histidine-tRNA ligase | 0.283 | 0.03712 |
| AP13ITG70254_s_at | pfam00332/Glycosyl hydrolases family 17 | 0.283 | 0.20357 |
| AP13ITG63167_at | PLN02655/ent-kaurene oxidase | 0.285 | 0.16187 |
| AP13ITG52645_at | Tol-pal system beta propeller repeat protein TolB | 0.286 | 0.31062 |
| AP13CTG14744_s_at | // | 0.287 | 0.17147 |
| AP13CTG24522_s_at | Catalytic domain of Protein Tyrosine Kinases | 0.287 | 0.22732 |
| KanlowCTG14711_s_at | PHA03100/ankyrin repeat protein | 0.288 | 0.15812 |
| AP13ITG40032_s_at | // | 0.290 | 0.00205 |
| AP13CTG11088_at | Transmembrane amino acid transporter protein | 0.291 | 0.23064 |
| AP13CTG02224_at | COG1199/Rad3-related DNA helicase | 0.292 | 0.04155 |
| AP13CTG70374_at | pfam00069/Protein kinase domain | 0.292 | 0.14869 |
| AP13ITG72285_at | // | 0.292 | 0.17089 |
| OTHSWSLT33230_s_at | A subfamily of OB folds | 0.292 | 0.00017 |
| KanlowCTG19923_s_at | PHA03247/large tegument protein UL36 | 0.293 | 0.06708 |
| KanlowCTG06220_s_at | // | 0.294 | 0.25602 |
| KanlowCTG24512_s_at | // | 0.294 | 0.17062 |
| OTHSWSLT34153_s_at | // | 0.294 | 0.00944 |
| AP13CTG27808_at | pfam00450/Serine carboxypeptidase | 0.295 | 0.12455 |
| AP13CTG18996_at | // | 0.295 | 0.07416 |
| AP13ITG63682-RC_at | // | 0.295 | 0.10560 |
| AP13ITG66335_at | PRK12678/transcription termination factor Rho | 0.296 | 0.01564 |
| AP13ITG54245_at | pfam00504/Chlorophyll A-B binding protein | 0.296 | 0.09274 |
| AP13CTG12869_at | Catalytic domain of Protein Kinase | 0.298 | 0.24375 |
| KanlowCTG47117_s_at | PLN02183/ferulate 5-hydroxylase | 0.299 | 0.19531 |
| KanlowCTG10974_s_at | Multi drug resistance-associated protein (MRP) | 0.299 | 0.00613 |
| AP13ITG44459_at | // | 0.301 | 0.06402 |
| OTHSWCTG05046_s_at | // | 0.302 | 0.25790 |
| AP13ITG41363_at | // | 0.302 | 0.00815 |
| AP13CTG27562_s_at | pfam00083/Sugar (and other) transporter | 0.302 | 0.06179 |
| AP13ITG72234_at | // | 0.302 | 0.16134 |
| AP13ITG73334_at | // | 0.303 | 0.07011 |
| AP13CTG58854_s_at | APETALA2 like DNA-binding domain protein | 0.304 | 0.02885 |
| OTHSWCTG03176_s_at | cd00180/Catalytic domain of Protein Kinase | 0.304 | 0.06248 |
| KanlowCTG16207_at | COG5147/Myb superfamily protein | 0.304 | 0.10464 |
| KanlowCTG08905_s_at | PLN02849/glycosyl hydrolase family 1 protein | 0.304 | 0.15842 |
| OTHSWCTG11457_at | cd00684/Plant Terpene Cyclases, Class 1 | 0.304 | 0.11881 |
| AP13CTG16311_at | cd03244/Domain 2 of the ABC subfamily C | 0.305 | 0.02688 |
| AP13ITG73202_at | // | 0.305 | 0.26632 |
| AlamoCTG12303_at | pfam02956/TT viral orf 1 | 0.305 | 0.12859 |
| AP13ITG41643_s_at | cd03244/Domain 2 of the ABC subfamily C | 0.305 | 0.08636 |
| AP13CTG29864_at | // | 0.306 | 0.04201 |
| KanlowCTG14963_s_at | TIGR00887/phosphate:H+ symporter | 0.306 | 0.12639 |
| KanlowCTG30431_at | cd00180/Catalytic domain of Protein Kinase | 0.306 | 0.15488 |
| KanlowCTG25218_at | Protein of unknown function (DUF2921) | 0.307 | 0.00003 |
| AP13ITG62309_at | cd06660/Aldo-keto reductase | 0.307 | 0.14400 |
| AP13ITG66986_at | Heat shock 70 kDa protein; Provisional | 0.307 | 0.13890 |
| AP13CTG50099_s_at | PRK09138/DNA topoisomerase I | 0.307 | 0.03136 |
| KanlowCTG40322_at | // | 0.307 | 0.01651 |
| AP13ITG54244_s_at | pfam00504/Chlorophyll A-B binding protein | 0.308 | 0.05128 |
| AP13CTG17588_s_at | TIGR00797/putative efflux protein | 0.309 | 0.02634 |
| AP13CTG27627_at | PLN00072/3-isopropylmalate isomerase | 0.310 | 0.00589 |
| AP13CTG27003_at | PLN02998/hydrolase | 0.310 | 0.22784 |
| AP13CTG30297_at | // | 0.310 | 0.00675 |
| KanlowSLT49511_at | pfam02956/TT viral orf 1 | 0.310 | 0.19267 |
| AlamoCTG04866_at | PLN02381/aminoacyl-tRNA ligase | 0.311 | 0.09904 |
| AlamoCTG09580_at | // | 0.312 | 0.00052 |
| AP13CTG11847_at | pfam03350/Uncharacterized protein family | 0.312 | 0.00684 |
| AP13CTG03072_at | // | 0.312 | 0.13511 |
| KanlowCTG21090_s_at | // | 0.312 | 0.00096 |
| KanlowCTG14868_s_at | pfam02338/OTU-like cysteine protease | 0.313 | 0.00656 |
| KanlowCTG02216_x_at | PHA03245/large tegument protein UL36 | 0.313 | 0.00114 |
| AP13CTG16311_s_at | cd03244/Domain 2 of the ABC subfamily C | 0.313 | 0.01107 |
| AP13ITG43615RC_at | Domain of unknown function (DUF2828) | 0.314 | 0.00544 |
| KanlowCTG23219_at | // | 0.315 | 0.01917 |
| OTHSWCTG17537_at | // | 0.315 | 0.03141 |
| KanlowCTG13984_x_at | PLN02183/ferulate 5-hydroxylase | 0.317 | 0.16906 |
| KanlowCTG17286_s_at | // | 0.317 | 0.01170 |
| AP13ITG41526_at | Leucine-rich repeat receptor-like protein kinase | 0.317 | 0.14308 |
| AP13ITG46767_s_at | // | 0.318 | 0.09075 |
| AP13.12746.m00013_s_at | smart00219/Tyrosine kinase, catalytic domain | 0.318 | 0.07023 |
| OTHSWCTG15870_s_at | pfam03106/WRKY DNA -binding domain | 0.318 | 0.12862 |
| OTHSWSLT27886_at | // | 0.320 | 0.19693 |
| AP13CTG10274_at | Transmembrane amino acid transporter protein | 0.320 | 0.10126 |
| AlamoCTG10010_at | // | 0.321 | 0.02326 |
| KanlowCTG44257_s_at | // | 0.321 | 0.05396 |
| AP13ITG76663RC_at | PHA03247/large tegument protein UL36 | 0.322 | 0.00145 |
| KanlowCTG07461_s_at | PHA03247/large tegument protein UL36 | 0.322 | 0.00942 |
| AP13CTG23895_at | cd03185/GST_C family, Class Tau subfamily | 0.323 | 0.19737 |
| AP13CTG13392_s_at | cd00180/Catalytic domain of Protein Kinases | 0.323 | 0.00001 |
| AlamoCTG03100_at | // | 0.323 | 0.00991 |
| AP13CTG16896_at | Transmembrane amino acid transporter protein | 0.324 | 0.10961 |
| AP13ITG66196_at | APETALA2 like DNA-binding domain protein | 0.324 | 0.02377 |
| AP13CTG54054_at | PHA03247/large tegument protein UL36 | 0.324 | 0.02085 |
| AP13ITG67646_at | cd03185/GST_C family | 0.324 | 0.00761 |
| AP13CTG58961_at | DNA polymerase III subunits gamma and tau | 0.324 | 0.10976 |
| AP13ITG70365_at | // | 0.325 | 0.05141 |
| AlamoCTG02782_at | cd01650/RT_nLTR | 0.325 | 0.01287 |
| AlamoCTG02352_at | // | 0.326 | 0.02229 |
| AP13ITG61604RC_at | PLN02650/dihydrokaempferol 4-reductase | 0.326 | 0.01565 |
| KanlowCTG20944_at | cd03249/MTABC3 | 0.326 | 0.13921 |
| KanlowCTG14326_s_at | PLN02795/allantoinase | 0.328 | 0.07929 |
| VS16ITG15279_at | // | 0.328 | 0.01502 |
| AP13ITG53164_s_at | // | 0.330 | 0.00012 |
| AP13CTG24874_s_at | TIGR00108/peptide chain release factor eRF/aRF | 0.330 | 0.00149 |
| AP13CTG29993_at | // | 0.331 | 0.00178 |
| AP13ITG68908_at | // | 0.331 | 0.15709 |
| KanlowCTG17154_s_at | pfam00069/Protein kinase domain | 0.331 | 0.12973 |
| AP13CTG06875_s_at | // | 0.332 | 0.01407 |
| AP13CTG28802_at | Pyridine nucleotide-disulphide oxidoreductase | 0.332 | 0.10104 |
| KanlowCTG16095_at | Membrane protein involved in colicin uptake | 0.332 | 0.00642 |
| AlamoCTG11374_s_at | cd00200/WD40 domain | 0.332 | 0.00109 |
| AP13ITG70516_at | smart00774/DNA binding domain | 0.333 | 0.19617 |
| KanlowCTG44074_s_at | TIGR00376/DNA helicase, putative | 0.333 | 0.04097 |
| OTHSWCTG07601_s_at | // | 0.335 | 0.00013 |
| KanlowCTG46528_s_at | pfam01554/MatE. The MatE domain | 0.335 | 0.04356 |
| OTHSWSLT23447_s_at | // | 0.337 | 0.04201 |
| AP13ITG68743_at | pfam02536/mTERF | 0.337 | 0.00916 |
| AP13ITG57354_at | pfam03169/OPT oligopeptide transporter protein | 0.338 | 0.03559 |
| AP13ITG63789_at | PRK12270/alpha-ketoglutarate decarboxylase | 0.340 | 0.01639 |
| AP13ITG50714_at | // | 0.340 | 0.04806 |
| AP13ITG39347_s_at | // | 0.341 | 0.02008 |
| KanlowCTG23104_s_at | TIGR00797/putative efflux protein, MATE family | 0.342 | 0.00766 |
| AP13ITG64316_at | // | 0.343 | 0.00251 |
| AP13CTG14667_at | DNA polymerase III subunits gamma and tau | 0.343 | 0.00684 |
| KanlowCTG38930_at | // | 0.343 | 0.00957 |
| AP13ITG41069_at | pfam01221/Dynein light chain type 1 | 0.344 | 0.03180 |
| AP13ITG57779_at | TIGR01642/U2 snRNP auxilliary factor | 0.345 | 0.02136 |
| KanlowSLT51921_at | Leucine-rich repeat receptor-like protein kinase | 0.346 | 0.00287 |
| KanlowCTG45028_s_at | // | 0.346 | 0.00112 |
| OTHSWCTG22293_at | pfam00139/Legume lectin domain | 0.346 | 0.00059 |
| KanlowCTG38984_s_at | DNA polymerase III subunits gamma and tau | 0.349 | 0.00627 |
| KanlowCTG31266_at | // | 0.350 | 0.01713 |
| AP13ITG46950_s_at | // | 0.351 | 0.00404 |
| OTHSWCTG14996_s_at | // | 0.351 | 0.04795 |
| KanlowCTG46799_s_at | PLN02893/cellulose synthase | 0.352 | 0.04829 |
| AlamoCTG12617_at | // | 0.353 | 0.04891 |
| AP13CTG36633_s_at | // | 0.353 | 0.00664 |
| AP13CTG39269_s_at | APETALA2 like DNA-binding domain protein | 0.353 | 0.00657 |
| AP13ITG38131_s_at | // | 0.353 | 0.00753 |
| KanlowCTG39835_s_at | PLN00014/light-harvesting-like protein 3 | 0.354 | 0.02097 |
| KanlowCTG21320_at | pfam00504/Chlorophyll A-B binding protein | 0.354 | 0.02197 |
| AP13CTG02031_s_at | TIGR00894/phosphate cotransporter | 0.354 | 0.00195 |
| KanlowCTG36135_at | TIGR01628/polyadenylate binding protein | 0.355 | 0.01801 |
| OTHSWCTG28918_s_at | // | 0.356 | 0.00420 |
| AP13CTG17588_at | TIGR00797/putative efflux protein, MATE family | 0.356 | 0.03076 |
| AlamoCTG02672_at | PLN02649/glucose-6-phosphate isomerase | 0.356 | 0.00380 |
| AP13CTG27593_at | TIGR01377/sarcosine oxidase | 0.357 | 0.02936 |
| OTHSWSLT34718_at | pfam05699/hAT family dimerization domain | 0.360 | 0.00531 |
| AP13ITG64615_at | PLN02534/UDP-glycosyltransferase | 0.363 | 0.00378 |
| KanlowCTG19343_s_at | COG0656/Aldo/keto reductase | 0.365 | 0.02176 |
| KanlowCTG47469_s_at | PLN02290/cytokinin trans-hydroxylase | 0.366 | 0.01454 |
| AP13CTG27627_s_at | PLN00072/3-isopropylmalate isomerase | 0.366 | 0.01280 |
| AlamoCTG07883_at | smart00717/SANT DNA-binding domain protein | 0.366 | 0.02563 |
| AP13CTG12747_at | // | 0.366 | 0.02136 |
| AP13CTG14872_at | // | 0.367 | 0.01511 |
| KanlowCTG17058_s_at | PLN00411/nodulin MtN21 family protein | 0.370 | 0.01351 |
| AlamoCTG10888_x_at | COG2226/Methylase | 0.370 | 0.04501 |
| AP13ITG63793_at | DNA polymerase III subunits gamma and tau | 0.370 | 0.00039 |
| AP13CTG14321_at | PLN02534/UDP-glycosyltransferase | 0.372 | 0.00837 |
| KanlowCTG39509_s_at | pfam01554/MatE | 0.373 | 0.00699 |
| OTHSWCTG07683_s_at | // | 0.375 | 0.04118 |
| KanlowCTG15986_s_at | DNA polymerase III subunits gamma and tau | 0.375 | 0.03785 |
| AP13CTG11236_at | TIGR00797/putative efflux protein | 0.375 | 0.01798 |
| AlamoCTG02212_s_at | // | 0.376 | 0.00667 |
| AP13ITG73600_s_at | // | 0.376 | 0.00858 |
| AP13CTG24859_at | // | 0.376 | 0.03934 |
| AP13ITG53182RC_at | PRK12270/alpha-ketoglutarate decarboxylase | 0.377 | 0.00967 |
| KanlowCTG33863_at | pfam04520/Protein of unknown function, DUF584 | 0.378 | 0.00888 |
| KanlowCTG32622_at | // | 0.379 | 0.01343 |
| OTHSWCTG19246_at | Leucine-rich repeat receptor-like protein kinase | 0.379 | 0.01037 |
| AP13CTG23306_at | PHA03247/large tegument protein UL36 | 0.379 | 0.04158 |
| KanlowCTG42074_s_at | cd06174/The Major Facilitator Superfamily (MFS) | 0.380 | 0.00734 |
| OTHSWCTG12138_s_at | // | 0.380 | 0.04494 |
| AP13ITG42003_s_at | COG0522/Ribosomal protein S4 and related protein | 0.381 | 0.00080 |
| AP13ITG57635_at | pfam10520/Kua-ubiquitin conjugating enzyme | 0.382 | 0.01464 |
| AP13ITG40116_at | pfam02365/No apical meristem (NAM) protein | 0.382 | 0.01062 |
| AP13ITG60726_at | smart00205/Thaumatin family | 0.382 | 0.02010 |
| AP13ITG72700_at | // | 0.382 | 0.03139 |
| AP13CTG17634_at | DNA polymerase III subunits gamma and tau | 0.383 | 0.00467 |
| KanlowCTG39504_s_at | DNA polymerase III subunits gamma and tau | 0.383 | 0.04361 |
| AP13CTG16495_at | cd00180/Catalytic domain of Protein Kinase | 0.384 | 0.01871 |
| AP13ITG55143_at | pfam02365/No apical meristem (NAM) protein | 0.385 | 0.00528 |
| AP13CTG12040_at | // | 0.385 | 0.00182 |
| KanlowCTG12457_x_at | TIGR00376/DNA helicase, putative | 0.386 | 0.03423 |
| AP13CTG09439_at | PLN00052/prolyl 4-hydroxylase | 0.387 | 0.03930 |
| AP13CTG22512_s_at | TIGR01640/F-box protein interaction domain | 0.387 | 0.00297 |
| OTHSWCTG13603_at | // | 0.387 | 0.03215 |
| AP13ITG73734_at | DNA polymerase III subunits gamma and tau | 0.387 | 0.00337 |
| AP13ITG40106_at | COG3964/Predicted amidohydrolase | 0.388 | 0.02287 |
| KanlowSLT50393_s_at | // | 0.388 | 0.02614 |
| AP13ITG69846_at | pfam00067/Cytochrome P450 | 0.388 | 0.00705 |
| AP13ITG75901_at | // | 0.388 | 0.00018 |
| KanlowCTG21090_at | // | 0.389 | 0.00928 |
| KanlowSLT56638_s_at | // | 0.389 | 0.01219 |
| KanlowCTG39045_at | Leucine-rich repeat receptor-like protein kinase | 0.389 | 0.00710 |
| KanlowCTG05584_s_at | Leucine-rich repeat receptor-like protein kinase | 0.391 | 0.01716 |
| AP13CTG27345_at | PHA03247/large tegument protein UL36 | 0.391 | 0.03474 |
| AP13ITG58411_s_at | pfam09713/Plant protein 1589 of unknown function | 0.392 | 0.00957 |
| AP13CTG59197_at | pfam02956/TT viral orf 1 | 0.392 | 0.00275 |
| AP13ITG60832_s_at | pfam00004/ATPase family | 0.392 | 0.03052 |
| AP13CTG15501_s_at | cd00839/metallophosphatase | 0.393 | 0.04276 |
| AP13CTG16324_at | pfam00190/Cupin | 0.393 | 0.00468 |
| OTHSWCTG08671_s_at | Multi drug resistance-associated protein (MRP) | 0.393 | 0.04764 |
| AP13CTG05211_at | // | 0.395 | 0.01638 |
| KanlowCTG29056_at | // | 0.395 | 0.03245 |
| AP13CTG11486_at | PHA03307/transcriptional regulator ICP4 | 0.396 | 0.03627 |
| AP13CTG21595_s_at | pfam06911/Senescence-associated protein | 0.396 | 0.00628 |
| AP13ITG58816_at | pfam03358/NADPH-dependent FMN reductase | 0.396 | 0.01041 |
| AP13CTG09069_at | cd03244/Domain 2 of the ABC subfamily C | 0.396 | 0.00178 |
| AP13CTG17814_at | PRK12438/hypothetical protein | 0.397 | 0.00025 |
| KanlowCTG04302_x_at | pfam00504/Chlorophyll A-B binding protein | 0.397 | 0.01383 |
| KanlowCTG18799_at | pfam02309/AUX/IAA family | 0.397 | 0.01400 |
| AP13CTG08616_at | pfam07847/Protein of unknown function (DUF1637) | 0.397 | 0.03003 |
| OTHSWCTG11990_x_at | PTZ00141/elongation factor 1 alpha | 0.399 | 0.00066 |
| AP13CTG12321_at | // | 0.399 | 0.00008 |
| AlamoCTG12828_at | // | 0.400 | 0.02811 |
| AlamoCTG04669_at | // | 0.400 | 0.02397 |
| AP13CTG11369-1_s_at | // | 0.400 | 0.01441 |
| KanlowCTG46894_s_at | // | 0.401 | 0.02880 |
| AP13CTG42772_s_at | // | 0.402 | 0.00326 |
| AP13CTG14468_at | COG0534/Na+-driven multidrug efflux pump | 0.403 | 0.00968 |
| KanlowCTG47572_s_at | APETALA2 like DNA-binding domain protein | 0.404 | 0.00303 |
| OTHSWCTG19610_s_at | // | 0.405 | 0.00429 |
| AP13ITG43492_s_at | PLN02687/flavonoid 3'-monooxygenase | 0.406 | 0.01252 |
| AP13CTG00379_s_at | cd01583/3-isopropylmalate dehydratase | 0.407 | 0.00214 |
| AP13ITG76100_at | cd00983/RecA | 0.407 | 0.00403 |
| AP13CTG59192_at | // | 0.407 | 0.02546 |
| KanlowCTG18961_at | cd00693/Horseradish peroxidase | 0.408 | 0.00083 |
| AP13CTG16931_s_at | // | 0.409 | 0.00250 |
| AP13CTG00624_s_at | // | 0.409 | 0.00127 |
| AP13CTG09391_at | pfam00931/NB-ARC domain | 0.411 | 0.00883 |
| AP13CTG05547_at | pfam02365/No apical meristem (NAM) protein | 0.412 | 0.04484 |
| OTHSWCTG13017_at | // | 0.412 | 0.01488 |
| AP13CTG06566_at | cd00200/WD40 domain | 0.412 | 0.04648 |
| AP13ITG37704_s_at | cd00038/the CAP family of transcription factor | 0.413 | 0.02353 |
| AP13CTG26505_at | // | 0.413 | 0.01118 |
| OTHSWSLT34912_at | // | 0.413 | 0.01273 |
| AP13CTG58694_s_at | // | 0.413 | 0.00182 |
| KanlowCTG29283_at | // | 0.414 | 0.01091 |
| AP13CTG27301_s_at | // | 0.414 | 0.03829 |
| AP13ITG67793_at | pfam00656/Caspase domain | 0.416 | 0.00048 |
| AP13ITG38519_at | PRK12678/transcription termination factor Rho | 0.416 | 0.01431 |
| AlamoCTG10988_s_at | // | 0.417 | 0.00009 |
| AP13ITG77378_at | // | 0.417 | 0.01125 |
| AlamoCTG05444_x_at | // | 0.417 | 0.02597 |
| AP13CTG08461_s_at | S1_CSL4: CSL4, S1-like RNA-binding domain | 0.418 | 0.00006 |
| KanlowCTG07471_s_at | Pathogenesis-related protein Bet v I family | 0.418 | 0.00006 |
| KanlowCTG34616_s_at | PRK12678/transcription termination factor Rho | 0.419 | 0.00657 |
| OTHSWCTG09737_x_at | // | 0.421 | 0.02900 |
| KanlowCTG40842_s_at | // | 0.421 | 0.02478 |
| AP13ITG69166_at | MTH00166/NADH dehydrogenase subunit 6 | 0.421 | 0.00975 |
| AP13CTG31837_at | // | 0.421 | 0.01443 |
| KanlowCTG23798_s_at | APETALA2 like DNA-binding domain protein | 0.421 | 0.01017 |
| AP13CTG29146_at | pfam03018/Dirigent-like protein | 0.421 | 0.00319 |
| AP13.12741.m00011_s_at | cd06899/legume lectin | 0.421 | 0.00822 |
| AP13CTG08394_at | pfam00012/Hsp70 protein | 0.422 | 0.00356 |
| AP13ITG58488_x_at | PHA02697/hypothetical protein | 0.423 | 0.00916 |
| AP13CTG16955RC_s_at | pfam06916/Protein of unknown function (DUF1279) | 0.423 | 0.00133 |
| OTHSWCTG06296_at | // | 0.423 | 0.04424 |
| AP13CTG27628_at | PLN00072/3-isopropylmalate isomerase | 0.425 | 0.01233 |
| AP13.12067.m00015_s_at | PLN00165/predicted protein | 0.425 | 0.00449 |
| AP13CTG28359_s_at | // | 0.428 | 0.04596 |
| AP13ITG58847_at | COG0484/DnaJ-class molecular chaperone | 0.428 | 0.00091 |
| AP13ITG60359_s_at | // | 0.428 | 0.00393 |
| AP13CTG07846_at | // | 0.429 | 0.00481 |
| AP13ITG58566_s_at | pfam04520/Protein of unknown function, DUF584 | 0.431 | 0.00638 |
| AP13CTG18371_s_at | pfam02365/No apical meristem (NAM) protein | 0.432 | 0.03436 |
| AP13ITG60705-RC_at | // | 0.432 | 0.01338 |
| KanlowCTG45154_s_at | COG5243/HRD ubiquitin ligase complex | 0.432 | 0.03983 |
| KanlowCTG22539_s_at | Arf GTPase Activating Protein | 0.433 | 0.00949 |
| AP13ITG39428_s_at | cd03236/The ATPase domain 1 of RNase L inhibitor | 0.434 | 0.00094 |
| AP13CTG01841_at | // | 0.434 | 0.00015 |
| KanlowCTG20088_s_at | Multi drug resistance-associated protein (MRP) | 0.434 | 0.00117 |
| AlamoCTG08558_at | Phosphoribosylformylglycinamidine cyclo-ligase | 0.435 | 0.04640 |
| KanlowCTG20128_s_at | pfam03514/GRAS family transcription factor | 0.435 | 0.03395 |
| KanlowCTG21568_at | Cinnamyl-alcohol dehydrogenase family protein | 0.435 | 0.01149 |
| AP13CTG17125RC_at | pfam00083/Sugar (and other) transporter | 0.436 | 0.04782 |
| AP13ITG63364_at | pfam01936/Protein of unknown function DUF88 | 0.436 | 0.00466 |
| AP13CTG12606_s_at | pfam00112/Papain family cysteine protease | 0.437 | 0.00072 |
| AP13ITG38235_s_at | // | 0.437 | 0.02076 |
| AP13CTG06456_at | TIGR01197/NRAMP metal ion transporter | 0.437 | 0.00241 |
| AP13ITG43614RC_s_at | pfam11443/Domain of unknown function (DUF2828) | 0.438 | 0.00610 |
| KanlowCTG15083_s_at | PHA03245/large tegument protein UL36 | 0.438 | 0.01408 |
| AP13CTG28386_at | pfam01397/Terpene synthase, N-terminal domain | 0.438 | 0.04918 |
| AP13CTG04179_s_at | // | 0.438 | 0.00334 |
| OTHSWCTG07252_s_at | COG2042/Uncharacterized conserved protein | 0.438 | 0.02753 |
| AP13CTG17141_at | PRK12323/DNA polymerase III subunits gamma and tau | 0.439 | 0.00407 |
| AP13CTG03381_at | cd00180/Catalytic domain of Protein Kinase | 0.440 | 0.03697 |
| KanlowCTG05535_s_at | PRK05306/translation initiation factor IF-2 | 0.440 | 0.04664 |
| AP13CTG08924-1_s_at | // | 0.440 | 0.02576 |
| OTHSWCTG11377_s_at | // | 0.441 | 0.00453 |
| AP13ITG62944_s_at | // | 0.441 | 0.03192 |
| AP13ITG33966_s_at | 3,8-divinyl protochlorophyllide a 8-vinyl reductase | 0.442 | 0.02091 |
| AlamoCTG08880_at | // | 0.442 | 0.00947 |
| AP13ITG53819_s_at | // | 0.442 | 0.00100 |
| AP13ITG44910_s_at | // | 0.443 | 0.02676 |
| OTHSWCTG19157_s_at | // | 0.444 | 0.00706 |
| AP13ITG52631_s_at | // | 0.444 | 0.00032 |
| KanlowSLT48969_s_at | // | 0.446 | 0.00171 |
| AP13CTG05735_at | cd00590/RRM (RNA recognition motif) | 0.447 | 0.00172 |
| KanlowCTG14042RC_at | pfam02956/TT viral orf 1. TT virus (TTV) | 0.447 | 0.02875 |
| AP13CTG01072_s_at | COG5271/AAA ATPase | 0.447 | 0.02826 |
| AlamoCTG11367_at | // | 0.448 | 0.00431 |
| AP13CTG10725_at | // | 0.449 | 0.01240 |
| AP13ITG60321_at | PHA03247/large tegument protein UL36 | 0.449 | 0.00147 |
| OTHSWCTG10497_at | // | 0.449 | 0.02388 |
| KanlowCTG42888_at | pfam05793/Transcription initiation factor IIF | 0.450 | 0.00128 |
| AP13CTG03272_s_at | pfam03962/Mnd1 family | 0.450 | 0.00017 |
| AP13CTG38854_s_at | cd03185/GST_C family | 0.450 | 0.04144 |
| AP13ITG65971_at | DNA polymerase III subunits gamma and tau | 0.451 | 0.01246 |
| KanlowCTG17636_x_at | pfam06507/Auxin response factor | 0.452 | 0.00033 |
| AP13ITG64115_at | smart00219/Tyrosine kinase, catalytic domain | 0.453 | 0.01791 |
| KanlowCTG23598_s_at | // | 0.453 | 0.03375 |
| AP13CTG15135_s_at | PLN03023/expansin-related protein 1 precursor | 0.453 | 0.01309 |
| AP13ITG64615_s_at | PLN02534/UDP-glycosyltransferase | 0.454 | 0.02811 |
| AP13ITG56189_at | pfam08212/Lipocalin-like domain | 0.458 | 0.02072 |
| AP13ITG47601_s_at | pfam01263/Aldose 1-epimerase | 0.458 | 0.00023 |
| OTHSWCTG11150_s_at | // | 0.460 | 0.00272 |
| AP13CTG23952RC_at | pfam02365/No apical meristem (NAM) protein | 0.460 | 0.02296 |
| KanlowCTG42433_s_at | Inositol 1, 3, 4-trisphosphate 5/6-kinase | 0.462 | 0.00613 |
| AP13ITG41126_s_at | PLN02954/phosphoserine phosphatase | 0.462 | 0.02517 |
| AP13ITG37279RC_at | // | 0.462 | 0.04824 |
| AP13ITG55990_s_at | // | 0.463 | 0.01001 |
| AP13CTG03166_at | // | 0.463 | 0.00930 |
| AP13CTG30476_at | // | 0.464 | 0.01359 |
| KanlowCTG02978_s_at | pfam02365/No apical meristem (NAM) protein | 0.464 | 0.01191 |
| AP13ITG45279_s_at | // | 0.465 | 0.01452 |
| OTHSWCTG12837_at | // | 0.465 | 0.00746 |
| AP13ITG40115_s_at | pfam02365/No apical meristem (NAM) protein | 0.466 | 0.01540 |
| AP13CTG31907_at | cd00180/Catalytic domain of Protein Kinase | 0.466 | 0.04259 |
| KanlowSLT56266_at | // | 0.466 | 0.00364 |
| KanlowCTG20048_at | // | 0.466 | 0.00464 |
| AP13CTG24555_at | PRK12678/transcription termination factor Rho | 0.466 | 0.00739 |
| AP13ITG69287_at | PRK12678/transcription termination factor Rho | 0.466 | 0.02679 |
| AP13ITG49260_s_at | PLN02441/cytokinin dehydrogenase | 0.466 | 0.00279 |
| AP13ITG39766_s_at | // | 0.467 | 0.04455 |
| AP13ITG63940-RC_at | // | 0.467 | 0.01197 |
| OTHSWCTG20305_at | Cinnamyl-alcohol dehydrogenase family protein | 0.468 | 0.02083 |
| KanlowCTG40575_s_at | // | 0.468 | 0.00056 |
| KanlowCTG29221_s_at | // | 0.468 | 0.04526 |
| AP13CTG27345_s_at | PHA03247/large tegument protein UL36 | 0.469 | 0.02701 |
| AP13ITG73134_s_at | // | 0.469 | 0.04704 |
| AP13ITG60014_s_at | cd03185/GST_C family, Class Tau subfamily | 0.469 | 0.02264 |
| AP13CTG17110_s_at | Domain of unknown function (DUF3411) | 0.470 | 0.00247 |
| KanlowCTG40467_s_at | 4-hydroxyphenylpyruvate dioxygenase | 0.471 | 0.03156 |
| OTHSWSLT37016_at | PLN02414/glycine dehydrogenase | 0.471 | 0.03089 |
| AP13CTG17402_at | Aspartate semialdehyde dehydrogenase | 0.474 | 0.04541 |
| AP13CTG17872_s_at | PRK00004/50S ribosomal protein L24 | 0.474 | 0.00137 |
| KanlowCTG45873_s_at | PLN02837/threonine-tRNA ligase | 0.475 | 0.00039 |
| AlamoCTG07404_at | // | 0.475 | 0.01128 |
| KanlowCTG00174_s_at | pfam01650/Peptidase C13 family | 0.475 | 0.02351 |
| AP13ITG68930_at | // | 0.476 | 0.02060 |
| KanlowCTG14333_s_at | pfam00067/Cytochrome P450 | 0.476 | 0.00074 |
| AP13CTG30253_at | PLN02205/trehalose-phosphatase | 0.476 | 0.03669 |
| OTHSWSLT39385_at | // | 0.476 | 0.00357 |
| AP13ITG68197_at | COG0590/Cytosine/adenosine deaminase | 0.477 | 0.00177 |
| AP13CTG09388_at | Phosphatidylinositol transfer protein | 0.477 | 0.01628 |
| AP13ITG34813_s_at | // | 0.477 | 0.04857 |
| OTHSWCTG12576_at | // | 0.477 | 0.00056 |
| KanlowCTG44172_at | // | 0.477 | 0.01639 |
| AP13CTG23694_at | pfam04770/ZF-HD protein dimerization region | 0.478 | 0.00656 |
| KanlowCTG26212RC_at | PLN02393/oxidoreductase | 0.480 | 0.01319 |
| AP13CTG08149_at | // | 0.480 | 0.00288 |
| KanlowCTG17945_at | S1_CSL4: CSL4, S1-like RNA-binding domain | 0.480 | 0.00266 |
| KanlowCTG11949_s_at | pfam02459/Adenoviral DNA terminal protein | 0.481 | 0.00191 |
| KanlowCTG43060_s_at | The Major Facilitator Superfamily (MFS) | 0.481 | 0.00162 |
| AP13ITG42775_at | pfam05839/Apc13p protein. | 0.482 | 0.02990 |
| AP13ITG41070_s_at | pfam01221/Dynein light chain type | 0.482 | 0.03887 |
| AP13CTG28750-RC_s_at | // | 0.483 | 0.00058 |
| AP13CTG06456_s_at | TIGR01197/NRAMP metal ion transporter | 0.484 | 0.03055 |
| KanlowSLT50884_s_at | PLN02500/steroid 22alpha-hydroxylase | 0.484 | 0.00533 |
| KanlowCTG32176_at | // | 0.484 | 0.01652 |
| KanlowCTG23582RC_at | DNA polymerase III subunits gamma and tau | 0.485 | 0.02444 |
| KanlowCTG37175_s_at | cd03190/GST_C family, ECM4-like subfamily | 0.485 | 0.03645 |
| AP13ITG64115_s_at | smart00219/Tyrosine kinase, catalytic domain | 0.485 | 0.01224 |
| KanlowCTG20143_at | Heavy metal translocating P-type ATPase | 0.486 | 0.02890 |
| AP13CTG13475_s_at | // | 0.486 | 0.02694 |
| KanlowCTG41694_s_at | PHA03247/large tegument protein UL36 | 0.486 | 0.00023 |
| VS16ITG12817_s_at | // | 0.486 | 0.01438 |
| AP13CTG21583_at | PLN00411/nodulin MtN21 family protein | 0.486 | 0.00807 |
| AP13CTG49907_s_at | // | 0.487 | 0.02108 |
| AP13CTG02557_at | pfam10440/WIYLD domain | 0.487 | 0.01609 |
| AP13CTG22449_at | Protein of unknown function (DUF1637) | 0.488 | 0.01161 |
| VS16ITG12817_at | // | 0.488 | 0.01957 |
| AP13CTG09862_at | TIGR01632/50S ribosomal protein L11 | 0.488 | 0.00392 |
| AP13CTG30907_s_at | pfam00060/Ligand-gated ion channel | 0.489 | 0.00365 |
| OTHSWSLT30311_at | // | 0.489 | 0.02178 |
| KanlowCTG37714_s_at | // | 0.489 | 0.04453 |
| AlamoCTG10882_at | // | 0.489 | 0.01056 |
| AP13CTG03280_s_at | Minichromosome maintenance protein | 0.489 | 0.00302 |
| AP13CTG59032_at | PLN00165/predicted protein | 0.490 | 0.00099 |
| AlamoCTG02332_s_at | // | 0.490 | 0.00179 |
| KanlowCTG41322_s_at | PLN02414/glycine dehydrogenase | 0.490 | 0.01643 |
| KanlowCTG33079_s_at | // | 0.490 | 0.04675 |
| KanlowCTG15276_s_at | // | 0.491 | 0.00167 |
| AP13CTG04636_s_at | PLN02972/histidine-tRNA ligase | 0.491 | 0.02081 |
| OTHSWSLT24983_s_at | pfam01738/Dienelactone hydrolase family | 0.491 | 0.00099 |
| AP13CTG12832_s_at | cd04897/ACT domain-containing protein | 0.491 | 0.04976 |
| AP13ITG61585_at | // | 0.491 | 0.00076 |
| AP13CTG26159_s_at | // | 0.491 | 0.00710 |
| OTHSWCTG17833_s_at | FAD binding domain of DNA photolyase | 0.491 | 0.02423 |
| AlamoCTG13341_s_at | // | 0.491 | 0.02028 |
| AP13CTG11844_at | Uncharacterized protein family (UPF0160) | 0.492 | 0.00180 |
| AP13CTG05671_at | TIGR00797/putative efflux protein, MATE family | 0.492 | 0.00888 |
| AP13CTG08614_at | pfam00069/Protein kinase domain | 0.492 | 0.04175 |
| AP13CTG00560_s_at | TIGR02917/TPR-repeat lipoprotein | 0.492 | 0.00449 |
| AP13ITG59727_at | // | 0.493 | 0.00106 |
| AP13ITG70794_at | // | 0.493 | 0.01085 |
| OTHSWCTG20305_s_at | Cinnamyl-alcohol dehydrogenase family protein | 0.493 | 0.03883 |
| AP13CTG73219_at | pfam00083/Sugar (and other) transporter | 0.494 | 0.03158 |
| AP13ITG35732_s_at | pfam03195/Protein of unknown function DUF260 | 0.494 | 0.01261 |
| AlamoCTG12279_s_at | pfam02956/TT viral orf 1. TT virus (TTV) | 0.494 | 0.01939 |
| AP13ITG74587-RC_at | // | 0.495 | 0.01894 |
| AP13ITG69649_at | PLN02687/flavonoid 3'-monooxygenase | 0.495 | 0.02757 |
| AP13ITG61498_s_at | // | 0.495 | 0.00200 |
| AP13CTG04179_at | // | 0.495 | 0.01658 |
| AP13ITG38338_s_at | DNA polymerase III subunits gamma and tau | 0.496 | 0.00542 |
| AP13CTG27575_at | TIGR00906/cationic amino acid transport permease | 0.496 | 0.00994 |
| AP13CTG15069_at | PHA03245/large tegument protein UL36 | 0.496 | 0.00036 |
| OTHSWCTG22807_at | // | 0.496 | 0.00621 |
| KanlowCTG20258_at | // | 0.497 | 0.02555 |
| AP13ITG53181_at | PRK12270/alpha-ketoglutarate decarboxylase | 0.498 | 0.02094 |
| OTHSWCTG12788_s_at | // | 0.498 | 0.00768 |
| AP13CTG29495_s_at | PyridoxaL 5'-Phosphate Dependent Enzymes class IV | 0.498 | 0.01005 |
| AP13CTG18599_at | PLN00145/tyrosine/nicotianamine aminotransferase | 0.498 | 0.00756 |
| AlamoCTG10704_at | COG4886/Leucine-rich repeat (LRR) protein | 0.498 | 0.00699 |
| AP13ITG58300_at | pfam04832/SOUL heme-binding protein | 0.498 | 0.01000 |
| AP13CTG15145_s_at | COG1095/DNA-directed RNA polymerase | 0.499 | 0.00071 |
| AP13CTG04892_s_at | smart00338/basic region leucin zipper | 0.499 | 0.03362 |
| AP13CTG07687_at | smart00219/Tyrosine kinase | 0.499 | 0.03953 |
| AP13ITG70038-RC_at | // | 0.499 | 0.00221 |
| AP13ITG60459_at | pfam12609/Wound-induced protein | 0.500 | 0.00945 |
| KanlowCTG08392_s_at | pfam00067/Cytochrome P450 | 0.500 | 0.02410 |
