# Supplementary material for: Overexpression of the WOX gene STENOFOLIA improves biomass yield and sugar release in transgenic grasses and display altered cytokinin homeostasis
Source: PLoS Genet. 2017 Mar 6;13(3):e1006649. doi: 10.1371/journal.pgen.1006649 (PMC5358894; doi:10.1371/journal.pgen.1006649)
Supplement: S4 Table — Fold change presented as relative abundance of transcript in STF overexpression/control (UBI::STF/UBI::GUS) switchgrass plants. P-value calculated as described in materials and methods. “//”, no significant similarity found. (DOC) [file pgen.1006649.s011.doc]

| **Probeset ID** | **CDD annotation** | **Fold change** | **P-value** |
| --- | --- | --- | --- |
| AP13CTG09031_s_at | PRK12678/transcription termination factor Rho | 50.118 | 0.37425 |
| KanlowCTG46495RC_s_at | PHA03247/large tegument protein UL36 | 11.876 | 0.35962 |
| AP13ITG57468-RC_at | // | 11.026 | 0.35238 |
| OTHSWCTG08878_s_at | pfam09713/Plant protein 1589 of unknown function | 10.577 | 0.02337 |
| AP13ITG69850_s_at | PLN00015/protochlorophyllide reductase | 10.387 | 0.04904 |
| AP13CTG06073_s_at | pfam09713/Plant protein 1589 of unknown function | 10.185 | 0.02848 |
| AP13ITG57467-RC_at | // | 9.518 | 0.33981 |
| AP13CTG40946_s_at | pfam09713/Plant protein 1589 of unknown function | 9.307 | 0.02561 |
| VS16ITG02633RC_x_at | PHA03247/large tegument protein UL36 | 8.237 | 0.38212 |
| AP13CTG11142_s_at | // | 8.015 | 0.38141 |
| AP13CTG06064RC_at | PLN02676/polyamine oxidase | 7.649 | 0.07537 |
| AP13CTG10402_s_at | cd00684/Plant Terpene Cyclases, Class 1 | 7.609 | 0.02815 |
| AP13CTG58880_at | PLN00015/protochlorophyllide reductase | 7.586 | 0.01418 |
| AP13ITG44267_s_at | PHA03247/large tegument protein UL36 | 6.646 | 0.37436 |
| KanlowCTG35008_s_at | cd07920/Pumilio-family RNA binding domain | 6.621 | 0.39194 |
| AP13CTG59177_at | pfam02469/Fasciclin domain | 6.452 | 0.17025 |
| AP13CTG52406_s_at | // | 6.426 | 0.37519 |
| AP13.12938.m00005_s_at | PTZ00183/centrin | 6.004 | 0.34202 |
| AP13ITG54760_at | Light-harvesting complex II chlorophyll-a/b binding protein | 5.615 | 0.00061 |
| AP13CTG22943_s_at | pfam00010/Helix-loop-helix DNA-binding domain | 5.386 | 0.00950 |
| AP13ITG52576_at | PLN02549/asparagine synthase | 5.344 | 0.24567 |
| AP13ITG64032_at | pfam03059/Nicotianamine synthase protein | 5.273 | 0.05461 |
| KanlowCTG39353_s_at | PLN02643/galactose-1-phosphate uridylyltransferase | 5.171 | 0.00155 |
| KanlowCTG06990_s_at | // | 4.990 | 0.23933 |
| KanlowCTG13566_s_at | PLN02687/flavonoid 3'-monooxygenase | 4.925 | 0.08213 |
| KanlowCTG24362_s_at | APETALA2 like DNA-binding domain protein | 4.766 | 0.27937 |
| KanlowCTG46500_s_at | PRK07764/DNA polymerase III subunits gamma and tau | 4.658 | 0.22026 |
| AP13CTG29532_s_at | PLN02549/asparagine synthase | 4.550 | 0.04875 |
| AP13CTG10860_s_at | pfam03168/Late embryogenesis abundant protein | 4.525 | 0.16242 |
| KanlowCTG13087_s_at | PLN02549/asparagine synthase | 4.460 | 0.03356 |
| AP13ITG52658_at | light-harvesting complex II chlorophyll-a/b binding protein | 4.446 | 0.04813 |
| KanlowCTG11572_s_at | PTZ00183/centrin | 4.363 | 0.30939 |
| AP13ITG70783_at | pfam00232/Glycosyl hydrolase family 1 | 4.352 | 0.03969 |
| AP13ITG65544_s_at | pfam05955/Equine herpesvirus glycoprotein gp2 | 4.270 | 0.33967 |
| AP13CTG15608_s_at | // | 4.239 | 0.12526 |
| KanlowCTG41562_at | // | 4.233 | 0.00260 |
| AP13CTG15691_s_at | pfam01190/Pollen protein | 4.177 | 0.08334 |
| AP13ITG52434_s_at | // | 4.149 | 0.00489 |
| AP13ITG73149_at | PLN02880/tyrosine decarboxylase | 4.090 | 0.34566 |
| AP13ITG74064_at | // | 4.073 | 0.31936 |
| KanlowCTG03580_at | pfam00232/Glycosyl hydrolase family 1 | 4.017 | 0.34702 |
| AP13ITG49631_s_at | cd01056/eukaryotic ferritin | 4.006 | 0.00033 |
| AP13ITG62380_at | pfam01190/Pollen protein | 3.994 | 0.08753 |
| AP13ITG69869_at | smart00835/Cupin | 3.921 | 0.26372 |
| KanlowCTG19830RC_s_at | // | 3.886 | 0.23238 |
| KanlowCTG04653_s_at | pfam06200/tify domain | 3.861 | 0.30958 |
| AP13ITG65716_at | cd00693/Horseradish peroxidase | 3.817 | 0.05530 |
| AP13CTG11250_s_at | // | 3.813 | 0.16715 |
| KanlowCTG13907_s_at | pfam01190/Pollen protein | 3.780 | 0.14883 |
| AP13ITG77101_at | pfam02956/TT viral orf 1 | 3.697 | 0.07764 |
| AP13ITG63765_at | // | 3.668 | 0.12264 |
| AP13ITG38950_at | pfam00280/Potato inhibitor I family | 3.667 | 0.10351 |
| AP13CTG17381_s_at | PLN02164/sulfotransferase family protein | 3.617 | 0.04579 |
| KanlowCTG01327_s_at | PLN02609/catalase | 3.555 | 0.01770 |
| KanlowCTG27550_s_at | // | 3.527 | 0.19430 |
| AP13ITG71045-RC_at | // | 3.520 | 0.32373 |
| AP13CTG12432_at | TIGR00815/high affinity sulphate transporter 1 | 3.488 | 0.38730 |
| AP13CTG31850_s_at | pfam00582/Universal stress protein family | 3.475 | 0.03982 |
| AP13CTG23747_s_at | pfam02298/Plastocyanin-like domain | 3.436 | 0.16270 |
| AP13CTG59151_at | TIGR01675/plant acid phosphatase | 3.422 | 0.05494 |
| AlamoCTG14050_s_at | TIGR00815/high affinity sulphate transporter | 3.382 | 0.30401 |
| KanlowCTG06613_s_at | pfam02956/TT viral orf 1. TT virus (TTV) | 3.382 | 0.09016 |
| AP13CTG53749_at | pfam00407/Pathogenesis-related protein Bet v I family | 3.339 | 0.06603 |
| AP13CTG19132_at | // | 3.313 | 0.14009 |
| KanlowCTG23726_at | COG0667/Predicted oxidoreductase | 3.311 | 0.06029 |
| AP13CTG14427_at | PLN02477/glutamate dehydrogenase | 3.299 | 0.17868 |
| AP13CTG15608_at | // | 3.291 | 0.18585 |
| AlamoCTG02211_s_at | // | 3.288 | 0.01075 |
| AP13CTG23271_s_at | pfam00280/Potato inhibitor I family | 3.288 | 0.11242 |
| AP13ITG52400_s_at | // | 3.233 | 0.35966 |
| AP13CTG34919_s_at | pfam02956/TT viral orf 1. TT virus (TTV) | 3.202 | 0.16520 |
| KanlowCTG24441_s_at | PTZ00074/60S ribosomal protein L34 | 3.192 | 0.30297 |
| AP13CTG27050_at | // | 3.185 | 0.00839 |
| AlamoCTG02469_s_at | // | 3.162 | 0.17028 |
| KanlowCTG46206_s_at | TIGR02997/RNA polymerase sigma factor | 3.155 | 0.03078 |
| AP13CTG02758-5_at | // | 3.147 | 0.00395 |
| AlamoCTG08015_at | // | 3.137 | 0.00253 |
| AP13CTG15767_at | PHA03247/large tegument protein UL36 | 3.125 | 0.13776 |
| KanlowCTG07696RC_at | pfam00450/Serine carboxypeptidase | 3.120 | 0.06383 |
| AP13ITG45283_s_at | smart00353/helix loop helix domain | 3.107 | 0.39030 |
| AP13CTG17381_at | PLN02164/sulfotransferase family protein | 3.103 | 0.04683 |
| AP13CTG02921_at | pfam00561/alpha/beta hydrolase fold | 3.096 | 0.10468 |
| KanlowCTG15509_s_at | // | 3.095 | 0.00362 |
| AP13ITG56721_at | smart00774/The WRKY domain transcription factor | 3.087 | 0.07065 |
| AP13ITG60632_at | pfam03018/Dirigent-like protein | 3.084 | 0.01829 |
| AP13ITG43286_s_at | pfam02956/TT viral orf 1. TT virus (TTV) | 3.084 | 0.10220 |
| KanlowCTG22494_s_at | TIGR01053/zinc finger domain, LSD1 subclass | 3.041 | 0.00106 |
| AP13ITG73777_at | PRK12678/transcription termination factor Rho | 3.036 | 0.00387 |
| AP13CTG38397_at | Phosphate-induced protein 1 conserved region | 3.026 | 0.29089 |
| AP13CTG07763_s_at | cd01056/eukaryotic ferritins | 3.022 | 0.00022 |
| OTHSWCTG20253_at | pfam05699/hAT family dimerization domain | 3.008 | 0.32699 |
| KanlowCTG14116_s_at | PLN02609/catalase | 2.997 | 0.00196 |
| AP13ITG55345_at | PLN00050/expansin A | 2.958 | 0.00913 |
| KanlowCTG07697_s_at | // | 2.939 | 0.04180 |
| AP13CTG31330_s_at | PTZ00318/NADH dehydrogenase | 2.928 | 0.02571 |
| AlamoSLT16515_s_at | PLN02609/catalase | 2.914 | 0.00543 |
| AP13CTG07574_s_at | PLN02336/phosphoethanolamine N-methyltransferase | 2.903 | 0.01917 |
| KanlowCTG00493_s_at | // | 2.880 | 0.00374 |
| KanlowCTG34417_at | PLN02609/catalase | 2.857 | 0.00879 |
| AP13CTG15216_at | pfam01048/Phosphorylase superfamily | 2.852 | 0.01652 |
| KanlowCTG12246_s_at | TIGR02997/RNA polymerase sigma factor | 2.843 | 0.00622 |
| KanlowCTG32144_s_at | // | 2.826 | 0.01083 |
| AP13ITG66127_at | // | 2.821 | 0.00864 |
| AP13ITG47641_s_at | // | 2.812 | 0.00772 |
| AP13ITG65927_at | pfam00232/Glycosyl hydrolase family | 2.775 | 0.04637 |
| KanlowCTG32993_s_at | pfam02431/Chalcone-flavanone isomerase | 2.773 | 0.04013 |
| AP13ITG66839_s_at | pfam02496/ABA/WDS induced protein | 2.749 | 0.00380 |
| AP13ITG39071_s_at | PLN00176/galactinol synthase | 2.723 | 0.04897 |
| AlamoCTG07057_s_at | PLN02998/hydrolase | 2.718 | 0.00594 |
| KanlowCTG08049_s_at | pfam00153/Mitochondrial carrier protein | 2.712 | 0.04507 |
| AP13CTG13664_s_at | PLN02955/8-amino-7-oxononanoate synthase | 2.698 | 0.04233 |
| AP13CTG08339_s_at | TIGR02169/chromosome segregation protein SMC | 2.689 | 0.00273 |
| AP13ITG44837_at | PLN02621/nicotinamidase | 2.645 | 0.01909 |
| AP13ITG56929_at | TIGR02008/ferredoxin [2Fe-2S] | 2.622 | 0.00297 |
| KanlowCTG19995RC_s_at | PLN02966/cytochrome P450, family 83, subfamily protein | 2.621 | 0.00042 |
| AP13ITG66508_at | // | 2.620 | 0.02396 |
| AP13CTG00753_at | // | 2.601 | 0.00529 |
| AP13ITG55469RC_at | pfam00332/Glycosyl hydrolases family 17 | 2.598 | 0.04976 |
| AP13CTG69025_at | pfam00334/Nucleoside diphosphate kinase | 2.585 | 0.03670 |
| AP13CTG24136_at | PRK12323/DNA polymerase III subunits gamma and tau | 2.578 | 0.00717 |
| AP13ITG69067_at | PRK12678/transcription termination factor Rho | 2.574 | 0.03807 |
| AP13CTG15834_at | TIGR02997/RNA polymerase sigma factor | 2.570 | 0.03995 |
| AP13CTG16557-1_at | // | 2.569 | 0.00620 |
| OTHSWCTG19810_s_at | pfam06943/LSD1 zinc finger | 2.563 | 0.01283 |
| AP13CTG09367_at | PHA03245/large tegument protein UL36 | 2.560 | 0.02370 |
| AP13CTG24068_s_at | // | 2.556 | 0.00974 |
| AP13ITG41769_s_at | COG5637/Predicted integral membrane protein | 2.552 | 0.02317 |
| OTHSWCTG11606_s_at | smart00271/DnaJ molecular chaperone homology domain | 2.550 | 0.02574 |
| AP13CTG26093_at | PLN02874/enoyl-CoA hydratase/isomerase family protein | 2.546 | 0.00633 |
| KanlowCTG09118_s_at | PHA03247/large tegument protein UL36 | 2.501 | 0.03140 |
| AP13ITG37902-RC_s_at | // | 2.483 | 0.00120 |
| AP13CTG22969_at | CHL00045/cytochrome c biogenesis protein | 2.481 | 0.00090 |
| AP13ITG55940-RC_at | // | 2.465 | 0.02514 |
| KanlowCTG13875_s_at | pfam03647/Transmembrane proteins 14C | 2.449 | 0.00378 |
| AP13ITG38578_at | // | 2.449 | 0.03905 |
| AP13ITG42745_at | pfam01150/GDA1/CD39 (nucleoside phosphatase) family | 2.448 | 0.04350 |
| AP13ITG36469_s_at | CHL00018/RNA polymerase beta' subunit | 2.445 | 0.04369 |
| AP13ITG63601_s_at | smart00704/CDGSH-type zinc finger. Function unknown | 2.443 | 0.00010 |
| KanlowCTG30896_s_at | PLN02459/adenylate kinase family protein | 2.410 | 0.00170 |
| AP13ITG44541_s_at | pfam00010/Helix-loop-helix DNA-binding domain | 2.405 | 0.01258 |
| AP13ITG59759-RC_at | // | 2.402 | 0.02249 |
| KanlowCTG10800_s_at | PLN02214/cinnamoyl-CoA reductase | 2.401 | 0.03278 |
| AP13ITG60331-RC_at | // | 2.400 | 0.02454 |
| AP13ITG64496_at | cd03185/GST_C family, Class Tau subfamily | 2.392 | 0.00712 |
| KanlowCTG20789_at | Cytochrome P450, family 94, subfamily C protein | 2.386 | 0.03870 |
| AP13ITG45616_at | // | 2.377 | 0.04785 |
| AP13ITG67774-RC_at | // | 2.373 | 0.02053 |
| AP13CTG11482_s_at | pfam06764/Protein of unknown function (DUF1223) | 2.359 | 0.01048 |
| AP13CTG09405_s_at | Ascorbate peroxidases and cytochrome C peroxidase | 2.345 | 0.03454 |
| AP13ITG43915_s_at | // | 2.342 | 0.03136 |
| KanlowCTG21277_s_at | // | 2.340 | 0.00005 |
| AP13ITG55594_x_at | PHA03245/large tegument protein UL36 | 2.338 | 0.00687 |
| AP13ITG69829_at | cd05476/Chroloplast Nucleoids DNA-binding Protease | 2.334 | 0.01232 |
| KanlowCTG19033_s_at | // | 2.325 | 0.01878 |
| AP13CTG55227_s_at | PHA03307/transcriptional regulator ICP4 | 2.322 | 0.00143 |
| AP13CTG27437_s_at | pfam06203/CCT motif | 2.319 | 0.04268 |
| AP13ITG69597_at | cd00317/cyclophilin | 2.311 | 0.02751 |
| AP13CTG51310_at | // | 2.297 | 0.04230 |
| AlamoCTG13287_s_at | pfam04515/Plasma-membrane choline transporter | 2.290 | 0.02016 |
| KanlowCTG45315_at | Polygalacturonate 4-alpha-galacturonosyltransferase | 2.285 | 0.01763 |
| AP13CTG01559-1_at | // | 2.277 | 0.01852 |
| AP13CTG27565_at | // | 2.276 | 0.02475 |
| KanlowCTG05802_s_at | Ascorbate peroxidases and cytochrome C peroxidase | 2.265 | 0.04915 |
| AP13ITG64033_at | pfam03018/Dirigent-like protein | 2.264 | 0.03852 |
| AP13CTG04617_at | smart00219/Tyrosine kinase, catalytic domain | 2.258 | 0.01407 |
| AP13CTG26987_at | PLN02336/phosphoethanolamine N-methyltransferase | 2.256 | 0.02145 |
| KanlowCTG04849_s_at | // | 2.252 | 0.00427 |
| AP13ITG56623_s_at | pfam03195/Protein of unknown function DUF260 | 2.244 | 0.03137 |
| AP13.11934.m00011_s_at | pfam03195/Protein of unknown function DUF260 | 2.241 | 0.01441 |
| AP13CTG10312_s_at | // | 2.240 | 0.03496 |
| AP13CTG23871_s_at | PHA03247/large tegument protein UL36 | 2.239 | 0.00526 |
| AP13CTG23999_at | cd00693/Horseradish peroxidase | 2.238 | 0.01053 |
| OTHSWSLT28522_at | // | 2.232 | 0.00369 |
| OTHSWCTG18240_s_at | // | 2.229 | 0.00878 |
| AP13ITG54529_s_at | COG1026/Predicted Zn-dependent peptidase | 2.220 | 0.01504 |
| KanlowCTG08482_s_at | // | 2.218 | 0.00493 |
| KanlowSLT49966_at | PLN00193/alpha-expansin | 2.196 | 0.04450 |
| AP13CTG15647_at | pfam10839/Protein of unknown function (DUF2647) | 2.193 | 0.01710 |
| KanlowCTG12693-1_s_at | // | 2.183 | 0.04582 |
| KanlowCTG14693_s_at | PLN02272/glyceraldehyde-3-phosphate dehydrogenase | 2.173 | 0.03601 |
| AP13CTG01561_at | pfam12552/Protein of unknown function (DUF3741) | 2.172 | 0.01293 |
| KanlowCTG03057_s_at | cd01837/SGNH_plant_lipase_like | 2.162 | 0.04377 |
| KanlowCTG13799_s_at | // | 2.159 | 0.00449 |
| AP13ITG43809_s_at | pfam03195/Protein of unknown function DUF260 | 2.158 | 0.00633 |
| KanlowCTG23300_at | Lipocalin / cytosolic fatty-acid binding protein | 2.153 | 0.00064 |
| AP13ITG63868_at | pfam04718/Mitochondrial ATP synthase g subunit | 2.142 | 0.02221 |
| AP13ITG55941-RC_s_at | // | 2.140 | 0.02508 |
| AlamoSLT16547_at | PHA02664/hypothetical protein | 2.136 | 0.04540 |
| AlamoCTG13207_s_at | // | 2.130 | 0.04589 |
| AP13ITG64340_at | // | 2.126 | 0.01730 |
| KanlowCTG16296_s_at | PRK07003/DNA polymerase III subunits gamma and tau | 2.123 | 0.00076 |
| KanlowCTG01327_at | PLN02609/catalase | 2.114 | 0.01985 |
| AP13ITG66993_at | pfam11493/Thylakoid soluble phosphoprotein TSP9 | 2.111 | 0.00098 |
| AP13ITG46032_s_at | 5'-AMP-activated protein kinase beta subunit | 2.110 | 0.02505 |
| AP13CTG17073_at | cd02176/Xyloglucan endotransglycosylase | 2.104 | 0.01682 |
| OTHSWCTG01295_s_at | pfam03179/Vacuolar (H+)-ATPase G subunit | 2.098 | 0.01460 |
| AP13ITG63432-RC_at | // | 2.097 | 0.00920 |
| AP13ITG65810_at | // | 2.095 | 0.01772 |
| AP13CTG30880_x_at | // | 2.091 | 0.00170 |
| AP13CTG09299_at | pfam05701/Plant protein of unknown function (DUF827) | 2.088 | 0.02137 |
| AP13CTG07811_s_at | pfam00061/Lipocalin | 2.082 | 0.00243 |
| AlamoSLT17645_at | // | 2.066 | 0.00273 |
| AP13ITG39131_s_at | PHA03247/large tegument protein UL36 | 2.057 | 0.02886 |
| AlamoCTG09620_s_at | // | 2.044 | 0.00019 |
| AP13CTG00023_s_at | pfam03151/Triose-phosphate Transporter family | 2.041 | 0.04087 |
| AP13CTG15649_at | pfam02431/Chalcone-flavanone isomerase | 2.040 | 0.02663 |
| AP13CTG01036_s_at | cd01837/SGNH_plant_lipase_like | 2.039 | 0.02056 |
| AP13ITG68352_at | PHA03245/large tegument protein UL36 | 2.038 | 0.00033 |
| AlamoCTG11900_at | // | 2.038 | 0.00176 |
| AP13ITG56127_x_at | pfam00235/Profilin | 2.034 | 0.01849 |
| KanlowCTG44575_s_at | pfam00011/Hsp20/alpha crystallin family | 2.030 | 0.02978 |
| AP13ITG75042_at | pfam05918/Apoptosis inhibitory protein 5 (API5) | 2.023 | 0.02546 |
| KanlowCTG41963_at | cd03244/Domain 2 of the ABC subfamily C | 2.023 | 0.01133 |
| AP13ITG43964RC_s_at | PLN02336/phosphoethanolamine N-methyltransferase | 2.018 | 0.01863 |
| KanlowCTG34183_x_at | PRK12270/alpha-ketoglutarate decarboxylase | 2.017 | 0.00168 |
| AP13CTG62834_at | COG1097/RNA-binding protein Rrp4 and related protein | 2.017 | 0.02584 |
| AP13CTG23940_at | smart00835/Cupin | 2.015 | 0.03655 |
| AP13ITG35962RC_s_at | cd00693/Horseradish peroxidase | 2.014 | 0.00421 |
| AP13ITG38704RC_s_at | PLN00050/expansin A | 2.007 | 0.00848 |
| AP13CTG15493_s_at | // | 2.006 | 0.02286 |
| AP13CTG02758-6_at | // | 2.004 | 0.00683 |
