# Supplementary material for: Overexpression of the WOX gene STENOFOLIA improves biomass yield and sugar release in transgenic grasses and display altered cytokinin homeostasis
Source: PLoS Genet. 2017 Mar 6;13(3):e1006649. doi: 10.1371/journal.pgen.1006649 (PMC5358894; doi:10.1371/journal.pgen.1006649)
Supplement: S5 Table — The top two leaf blades of three UBI::STF transgenic rice lines and wild type at vegetative stage (2 months after planting) were collected for Quantification of Cytokinins. iP, isopentenyladenine; iPR, iP riboside; iP9G, iP 9-glucoside; tZ, zeatin; tZR, zeatin riboside; tZ9G, zeatin 9-glucoside. The unit is ng·g-1 FW. (DOC) [file pgen.1006649.s012.doc]

| **Cytokinin** | **iP** | **iPR** | **iP9G** | **tZ** | **tZR** | **tZ9G** |
| --- | --- | --- | --- | --- | --- | --- |
| **Control** | 0.180  0.142  0.164 | 0.188  0.223  0.244 | 0.752  0.738  0.784 | 0.160  0.136  0.134 | 0.057  0.056  0.059 | 0.546  0.492  0.463 |
| ***UBI::STF*** | 0.213  0.237  0.191 | 0.361  0.389  0.433 | 0.856  0.810  0.875 | 0.177  0.173  0.195 | 0.046  0.052  0.049 | 0.688  0.686  0.866 |
