# Supplementary material for: Overexpression of the WOX gene STENOFOLIA improves biomass yield and sugar release in transgenic grasses and display altered cytokinin homeostasis
Source: PLoS Genet. 2017 Mar 6;13(3):e1006649. doi: 10.1371/journal.pgen.1006649 (PMC5358894; doi:10.1371/journal.pgen.1006649)
Supplement: S6 Table — (DOC) [file pgen.1006649.s013.doc]

| **Gene name** | **Sequence ID** | **Organism** |
| --- | --- | --- |
| *OsCKX1* | [XM_015780365.1](https://www.ncbi.nlm.nih.gov/nucleotide/1002228191?report=genbank&log$=nuclalign&blast_rank=1&RID=0KAGJM0Z014) | Rice |
| *OsCKX2* | [XM_015773930.1](https://www.ncbi.nlm.nih.gov/nucleotide/1002226931?report=genbank&log$=nuclalign&blast_rank=1&RID=0KAH2RER015) | Rice |
| *OsCKX3* | [XM_015757560.1](https://www.ncbi.nlm.nih.gov/nucleotide/1002299275?report=genbank&log$=nuclalign&blast_rank=1&RID=0KAN1NJ4015) | Rice |
| *OsCKX4*  *OsCKX5* | [XM_015765857.1](https://www.ncbi.nlm.nih.gov/nucleotide/1002234264?report=genbank&log$=nuclalign&blast_rank=1&RID=0KAJ04X8015)  [XM_015770438.1](https://www.ncbi.nlm.nih.gov/nucleotide/1002226251?report=genbank&log$=nuclalign&blast_rank=1&RID=0KAHJAEG014) | Rice  Rice |
| *OsCKX6* | [XM_015769432.1](https://www.ncbi.nlm.nih.gov/nucleotide/1002241243?report=genbank&log$=nuclalign&blast_rank=2&RID=0KAJJZKP015) | Rice |
| *OsCKX7* | [XM_015768875.1](https://www.ncbi.nlm.nih.gov/nucleotide/1002240204?report=genbank&log$=nuclalign&blast_rank=1&RID=0KAJJZKP015) | Rice |
| *OsCKX8* | [XM_015778031.1](https://www.ncbi.nlm.nih.gov/nucleotide/1002258366?report=genbank&log$=nuclalign&blast_rank=1&RID=0KAK2UYG015) | Rice |
| *OsCKX9* | [XM_015783650.1](https://www.ncbi.nlm.nih.gov/nucleotide/1002269591?report=genbank&log$=nuclalign&blast_rank=1&RID=0KAKG1DG015) | Rice |
| *OsCKX10* | [XM_015786878.1](https://www.ncbi.nlm.nih.gov/nucleotide/1002276043?report=genbank&log$=nuclalign&blast_rank=1&RID=0KAKZFW3014) | Rice |
| *OsCKX11* | [XM_015795175.1](https://www.ncbi.nlm.nih.gov/nucleotide/1002292616?report=genbank&log$=nuclalign&blast_rank=1&RID=0KAME03V015) | Rice |
| *BdCKX1* | [XM_003565400.2](https://www.ncbi.nlm.nih.gov/nucleotide/721632396?report=genbank&log$=nuclalign&blast_rank=1&RID=0KBTMRDX014) | Brachypodium |
| *BdCKX2*  *BdCKX3*  *BdCKX4*  *BdCKX5*  *BdCKX6*  *BdCKX7*  *BdCKX8*  *BdCKX9*  *BdCKX10*  *BdCKX11*  *PvCKX1a*  *PvCKX1b*  *PvCKX4a*  *PvCKX4b*  *PvCKX5*  *PvCKX6*  *PvCKX9*  *PvCKX10*  *PvCKX11* | [XM_003564942.3](https://www.ncbi.nlm.nih.gov/nucleotide/960464941?report=genbank&log$=nuclalign&blast_rank=1&RID=0KBVRUXE014)  [XM_003571850.3](https://www.ncbi.nlm.nih.gov/nucleotide/960475074?report=genbank&log$=nuclalign&blast_rank=1&RID=0KC0YGBU015)  [XM_003565002.3](https://www.ncbi.nlm.nih.gov/nucleotide/960470214?report=genbank&log$=nuclalign&blast_rank=1&RID=0KC28UEM014)  [XM_003569888.2](https://www.ncbi.nlm.nih.gov/nucleotide/721645054?report=genbank&log$=nuclalign&blast_rank=1&RID=0KC3EH72014)  [XM_003571671.2](https://www.ncbi.nlm.nih.gov/nucleotide/960475916?report=genbank&log$=nuclalign&blast_rank=1&RID=0KC6RPH7014)  [XM_014900434.1](https://www.ncbi.nlm.nih.gov/nucleotide/960475914?report=genbank&log$=nuclalign&blast_rank=1&RID=0KC8BN27014)  [XM_014895765.1](https://www.ncbi.nlm.nih.gov/nucleotide/960492230?report=genbank&log$=nuclalign&blast_rank=1&RID=0KCG3WF4014)  [XM_010233170.2](https://www.ncbi.nlm.nih.gov/nucleotide/960464461?report=genbank&log$=nuclalign&blast_rank=1&RID=0KCH81XK015)  [XM_010229283.1](https://www.ncbi.nlm.nih.gov/nucleotide/721615215?report=genbank&log$=nuclalign&blast_rank=1&RID=0KCJFV9U015)  [XM_010236896.2](https://www.ncbi.nlm.nih.gov/nucleotide/960481852?report=genbank&log$=nuclalign&blast_rank=1&RID=0KCM57C6014)  Pavirv00059528m  Pavirv00035115m  Pavirv00028994m  Pavirv00052903m  Pavirv00007674m  Pavirv00027542m  Pavirv00060424m  Pavirv00010010m  Pavirv00025103m | Brachypodium  Brachypodium  Brachypodium  Brachypodium  Brachypodium  Brachypodium  Brachypodium  Brachypodium  Brachypodium  Brachypodium  Switchgrass  Switchgrass  Switchgrass  Switchgrass  Switchgrass  Switchgrass  Switchgrass  Switchgrass  Switchgrass |
