# Supplementary material for: Overexpression of the WOX gene STENOFOLIA improves biomass yield and sugar release in transgenic grasses and display altered cytokinin homeostasis
Source: PLoS Genet. 2017 Mar 6;13(3):e1006649. doi: 10.1371/journal.pgen.1006649 (PMC5358894; doi:10.1371/journal.pgen.1006649)
Supplement: S7 Table — (DOC) [file pgen.1006649.s014.doc]

| **Primer name** | **Sequence** |
| --- | --- |
| **For overexpression** |  |
| STF-attB1 | ggggacaagtttgtacaaaaaagcaggcttcATGTGGATGGTGGGTTACAATG |
| STF-attB2 | ggggaccactttgtacaagaaagctgggtcTCAGTTTTTCAAGGGAAGAAACTC |
| GFP-attB1 | ggggacaagtttgtacaaaaaagcaggcttcATGGGTAAAGGAGAACTTTTCA |
| GFP-F | ATGGGTAAAGGAGAACTTTTCA |
| STF-R-6*Gly | GCCACCCCCT CCGCCACC GTTTTTCAAGGGAAGAAACTCA |
| 6*Gly-F | GGTGGCGG AGGGGGTG |
| 2*HA R+TGA | TCAGGCATAATCA GGCACATCG |
| 2*HA+TGA-attB2 | ggggaccactttgtacaagaaagctgggtcTCAGGCATAATCA GGCACATCG |
| **For Luciferase assay** |  |
| proOsCKX9 XmaIF | cccccgggGTTCCTGTAAATTTCTGGTTGGTGGA |
| proOsCKX9 SacIR | cgagctcGGCTCTTCTACGTGTCAAGAATGAACAATAT |
| proOsCKX11 HindIIIF | ccaagcttCCTAAAGATGAGCAGGGACAGT |
| proOsCKX11 SacIR | cgagctcCCGCTGCGTTTCTTGTTTCT |
| proBdCKX11 XmaIF | cccccgggCCCCTTCTTCTAGCACCGCCAAG |
| proBdCKX11 BamHIR | cgggatccCTTGACCTGGCAAGCGGAGCGCGT |
| proPvCKX4b PstIF | aactgcagGCCTCCATCCTCCCTCTCTTGGG |
| proPvCKX4b XmaIR | cccccgggGGTGGCCGGCCGGCCGGCTGGCTCTT |
| STF-DEL-attB2 | ggggaccactttgtacaagaaagctgggtcTCAACTAGTATTTAAGTGGATAAAAG |
| **For DNA binding assay** |  |
| STF-BamHIF | cgggatccATGTGGATGGTGGGTTACAAT |
| STF-HM EcoRIR | cggaattcTCAATCGAATTGGAGCCATCC |
| STF-boxs-F | TCACTCATTAGGGCAAACTGTGGGTCATAATCAGGAACTAGGAGA |
|  | GTGG |
| STF-box1-R | AGGGCCTGAGATCGGCTTGTCATCGCGCATGCAAAAGAGCTCACG |
|  | TGAGCCCACTCTCCTAGTTCCTGAT |
| STF-box2-R | AGGGCCTGAGATCGGCTTGTCATCGCGCATGCAAAAGAGCTCAGC |
|  | TGAGCCCACTCTCCTAGTTCCTGAT |
| STF-box3-R | AGGGCCTGAGATCGGCTTGTCATCGCGCATGCAAAAGAGCATTGG |
|  | CCCACTCTCCTAGTTCCTGAT |
| STF-box4-R | AGGGCCTGAGATCGGCTTGTCATCGCGCATGCAAAAGAGCCCATT |
|  | AAGCCCACTCTCCTAGTTCCTGAT |
| STF-box-qF | CATTAGGGCAAACTGTGGGTC |
| STF-box-qR | CCTGAGATCGGCTTGTCATC |
| **For qRT-PCR** |  |
| STF-F | AATGAATCTGATCAAACCCTTCAAC |
| STF-R | TGCATTGATTGCTGAAGCTGATAT |
| OsActin-F | TCCATCTTGGCATCTCTCAG |
| OsActin-R | GTACCCGCATCAGGCATCTG |
| BdUBC18-F | GTCACCCGCAATGACTGTAAGTTC |
| BdUBC18-R | TTGTCTTGCGGACGTTGCTTTG |
| PvUbiquitin-F | TTCGTGGTGGCCAGTAAG |
| PvUbiquitin-R | AGAGACCAGAAGACCCAGGTACAG |
| OsCKX1-F | GCACCCGTGGCTCAACCTG |
| OsCKX1-R | GATGTCGGTGGCCGTCTGG |
| OsCKX2-F | TGTCCCTTCTACAATGGTGC |
| OsCKX2-R | CATCCTGACCTGCTCTTGCT |
| OsCKX3-F | TTTCTTATGCTGATGTGGGTG |
| OsCKX3-R | GAACATTGCTAATCTGAGGTCC |
| OsCKX4-F | TTCTACCTGGTTGGGTTCCT |
| OsCKX4-R | TGGGCTTTCCACTGCTTCT |
| OsCKX5-F | GCACGCAAAGAAACACGG |
| OsCKX5-R | ACAGTCGAGATGAGGCAGTAG |
| OsCKX6-F | GTTCTATGTCGTTGGGCTACTC |
| OsCKX6-R | TTGTGATGCGTAGTGTGGTAG |
| OsCKX7-F | GTGAGGGAGGAAGAAAGGG |
| OsCKX7-R | TGAGGATGACGCCAACAGG |
| OsCKX8-F | TGGAGGTGCTGGAGGAGTG |
| OsCKX8-R | CGCCTACGGTGAGGTACAAG |
| OsCKX9-F | GCCTTCCTCCTTATTCCACA |
| OsCKX9-R | TGGGACCATTGTTACTGTCTTTC |
| OsCKX10-F | CGTCCTACCTTCCCTGCTG |
| OsCKX10-R | GACTGGTATGGTGACCGTGAT |
| OsCKX11-F | GCTCTGGGAGGAGGTGCT |
| OsCKX11-R | CGGTGAGGCGGAGGTAGT |
| Os01g0835900-qF | CGACAACATCCAGGGCATCA |
| Os01g0835900-qR | GTAGATCAGCCCGGAGATGC |
| Os07g0549900-qF | GCATCTCGGGGCTCATCTAC |
| Os07g0549900-qR | GTGCTCGGTGTAGGTGACG |
| BdCKX1-F | GACAAGTACGACCCCAAGAA |
| BdCKX1-R | GGAGGTTTTCGCACACTTTTG |
| BdCKX2-F | ACAACGACAACCACATCACC |
| BdCKX2-R | TCTTTTCGTCCCACTTGTCC |
| BdCKX3-F | AGGTAATGTACGTGGTTGGC |
| BdCKX3-R | GTACTGTTTTGCTCCAATGCG |
| BdCKX4-F | TGACAGCATGGAACAGGAAG |
| BdCKX4-R | ATCGGTGTGGAAAAGAGTGG |
| BdCKX5-F | ACTACCTCTACCTGTCCGTG |
| BdCKX5-R | GCTCGTAGACATTACTGATCTGG |
| BdCKX6-F | TGTACCCCATGAACACCAAC |
| BdCKX6-R | AAGACTCACGGCATAGAACAC |
| BdCKX7-F | GTGGCTGTGGGAGATGTG |
| BdCKX7-R | TGTGTAATGTGGCAGGTACTG |
| BdCKX8-F | ATGGTTCTGAACGAGCACTC |
| BdCKX8-R | GTCTGGGCTGAAATTGATGTG |
| BdCKX9-F | ACAGACTACCTCCACCTTACAG |
| BdCKX9-R | CACCTCTTCCAGTCACAATCTC |
| BdCKX10-F | CTTGTTCTCCACCGCCAG |
| BdCKX10-R | TGTGTCCGAAATCCAAAGAGG |
| BdCKX11-F | GTGGCTCAACCTCTTCATCTC |
| BdCKX11-R | GGTCCCACTTGCTGTTGAG |
| Bradi2g22991-qF | GAGGGAAGGGAGGTAAGGGG |
| Bradi2g22991-qR | GGTGATGCCCTGGATGTTGT |
| Bradi1g68190-qF | AAGGTTCTGCGGGACAACAT |
| Bradi1g68190-qR | GTAGATGAGCCCCGAGATGC |
| PvCKX1a-F | CCACCCTCAACTACGACAAC |
| PvCKX1a-R | GAACTCCACGTACGCCAC |
| PvCKX1b-F | ACTACCTCTACCTCACCATCG |
| PvCKX1b-R | CTCGAACACGTTAGATATCTGGG |
| PvCKX4a-F | ACTATCTCCATCTCACGGTCG |
| PvCKX4a-R | CTCTTCCTGTCACAATCTCCAG |
| PvCKX4b-F | CTAGAGTTCTTGGACAGGGTG |
| PvCKX4b-R | TCTTTCAGGATCTTGCCGAAG |
| PvCKX5-F | ACTACCTCTACCTGTCCGTG |
| PvCKX5-R | CTCGTAGACATTGCTGATCTGG |
| PvCKX6-F | TGAGGAAGTTTTCTACGCCG |
| PvCKX6-R | CACTGACTCGTTCACCTTCTC |
| PvCKX9-F | GTCACCTACATCGAATTCCTGG |
| PvCKX9-R | TTTTCCCAAAGACCTCCCG |
| PvCKX10-F | CTTCCGTTTCCCTAGCAGATG |
| PvCKX10-R | GGTGAGGTGGGATGGAATAC |
| PvCKX11-F | GCTCAACCTCTTCGTCTCC |
| PvCKX11-R | GGTCCCACTTGCTCTTGAG |
| Pavirv00047620m-F | TTGCTTGTTCGTTTGGGTTAC |
| Pavirv00047620m-R | GAACAAAAGACAGCACAGATACG |
| Pavirv00049788m-F | TGGTGTTAGGGTTAGCAATGG |
| Pavirv00049788m-R | GCACAAAACAGACAAGGATACAC |
| AP13ITG38704RC_s_at-F | TGTGTAGCCTTGGATTATGGG |
| AP13ITG38704RC_s_at-R | CAAGGTGCCATCGGTGATAG |
| KanlowSLT49966_at-F | CTTGCATGCCATGTTACGTG |
| KanlowSLT49966_at-R | GTTCATCTAGGACACGGCAC |
| Medtr1g015410 F | GGTGTCATCACTAGAGCCAGAATCG |
| Medtr1g015410 R | CCCTCAACAAATCTCAACCCTCCAA |
| Medtr2g039340 F | TATGCCGTTCTTGGAGGACTAGGT |
| Medtr2g039340 R | GCTCTAGGACATAGGTGATGCCATA |
| Medtr2g039360 F | GATTTAGGTTCTCAGGTGAGACTCTATA |
| Medtr2g039360 R | CCCTTGCCCTGGTGACAATAT |
| Medtr2g039410 F | GAATAGCTCTTGAGCCAGCACCAA |
| Medtr2g039410 R | GCTTCAACTCTCCACTTCTCACTCT |
| Medtr3g036100 F | TCCCATCAACACTTTTTCAAACAGA |
| Medtr3g036100 R | CAGGTCCACTGGATGAGGCA |
| Medtr4g044110 F | GTGGATGTCTCTGGTGGTGAATTGT |
| Medtr4g044110 R | TGGAAGGTGCTGGTTCAAGAAGAAT |
| Medtr7g090920 F | AGGACTTGGTCAGTTTGGCATT |
| Medtr7g090920 R | CCTTTTGAACGCAATTTGACCT |
| MtActin-F | TCAATGTGCCTGCCATGTATGT |
| MtActin-R | ACTCACACCGTCACCAGAATCC |
| **For CHIP assay** |  |
| pOsCKX9-P1-qF | AGCAGCACATCCTCACATGG |
| pOsCKX9-P1-qR | GGTATGTATGTGGACTGGTGCA |
| pOsCKX9-P2-qF | TGGAAAACAGGCCCCAACAT |
| pOsCKX9-P2-qR | CAGGCCTGCTTGATCCCTC |
| pOsCKX9-P3-qF | TGTTCACATTGCCTTCAGACAG |
| pOsCKX9-P3-qR | GCCCACATGCATGCTGTTG |
| pOsCKX9-P4-qF | CACCATGCCTGAATCCCGAT |
| pOsCKX9-P4-qR  pOsCKX9-P5-qF  pOsCKX9-P5-qR | AGCAAAGTGGAAATGAGGGGA  TTCTGGTTGGTGGACAGGTC  CCCCTAAGGTCTGAACAACAAA |
| pOsCKX9-P6-qF  pOsCKX9-P6-qR  pOsCKX9-P7-qF  pOsCKX9-P7-qR  pOsCKX9-P8-qF  pOsCKX9-P8-qR  pOsCKX9-P9-qF  pOsCKX9-P9-qR | GTGAATGAAGGCCTTAGTTGCA  ACACCAAGATAACCGAGCGA  TCCCCAACCCATCCTATCGA  TCCTTCCAATTTCCAACGTGT  GCTGTGGGAATTGACGACAG  TGTATACAGGCACCTTTGATGG  GACCTTTAAATCCATAGCCTCGT  AGCATGAAACTTGAAGCAGCT |
| pOsCKX11-P1-qF | CCCAGGACCCAAACTACCAC |
| pOsCKX11-P1-qR | CCGCTGCGTTTCTTGTTTCT |
| pOsCKX11-P2-qF | CGCAATAGTGGCAGACAAGC |
| pOsCKX11-P2-qR | TTTGCATCTCCTCTCCCTGC |
| pOsCKX11-P3-qF | CGAAGGAAAACGACACGATCA |
| pOsCKX11-P3-qR | GTGAGTGAGACTGTGGGCTG |
| pOsCKX11-P4-qF | AGACCATCGACAACTGCACA |
| pOsCKX11-P4-qR | TGAGCAAAACCGTGAGACCT |
| pOsCKX11-P5-qF  pOsCKX11-P5-qR | CCTAAAGATGAGCAGGGACAGT  TCTGATGTGGACTTGCAGAGG |
| pOsCKX11-P6-qF  pOsCKX11-P6-qR  pOsCKX11-P7-qF  pOsCKX11-P7-qR  pOsCKX11-P8-qF  pOsCKX11-P8-qR  pOsCKX11-P9-qF  pOsCKX11-P9-qR  pOsCKX11-P10-qF  pOsCKX11-P10-qR  pOsCKX11-P11-qF pOsCKX11-P11-qR  pOsCKX11-P12-qF  pOsCKX11-P12-qR | TGATGAGCTCACCACGTTGT  TTTTGGTGCCCTGACATCTCA  CTCGGAGTCTGGATCTTGCC  TGGTCATTTCCCCGTGGTTT  ATCGTCCATGGTTCTGTCAGT  CGAGTGCACCTACGACTAGC  GCTCTGGGAGGAGGTGCT  CGGTGAGGCGGAGGTAGT  ACTCTCTCCTCGCTTCCTCG  CGGCGAAACAGAATAAGCGA  GACAAGATTCCCGCCGGTG  AAAGAGACGTTTCCCTCCGC  ATGTTTCCTGGCTGTCACGG  TCCACAGCAATCGACTGGAC |
| OsActin-qF | CCGGTAGAAGATGGCTGACG |
| OsActin-qR | CCCAGCCTTGACCATACCAG |
